# Evolution of the bovid cranium: morphological diversification under allometric constraint

**DOI:** 10.1038/s42003-021-02877-6

**Published:** 2022-01-19

**Authors:** Faysal Bibi, Joshua Tyler

**Affiliations:** 1 Museum für Naturkunde, Leibniz Institute for Evolution & Biodiversity Science, Invalidenstr. 43, Berlin, 10115 Germany; 2 Present Address: Milner Centre for Evolution, Department of Biology and Biochemistry, University of Bath, Bath, BA2 7AY UK

**Keywords:** Evolution, Zoology

## Abstract

The role of environmental selection in generating novel morphology is often taken for granted, and morphology is generally assumed to be adaptive. Bovids (antelopes and relatives) are widely differentiated in their dietary and climatic preferences, and presumably their cranial morphologies are the result of adaptation to different environmental pressures. In order to test these ideas, we performed 3D geometric morphometric analyses on 141 crania representing 96 bovid species in order to assess the influence of both extrinsic (e.g. diet, habitat) and intrinsic (size, modularity) factors on cranial shape. Surprisingly, we find that bovid crania are highly clumped in morphospace, with a large number of ecologically disparate species occupying a very similar range of morphology clustered around the mean shape. Differences in shape among dietary, habitat, and net primary productivity categories are largely non-significant, but we found a strong interaction between size and diet in explaining shape. We furthermore found no evidence for modularity having played a role in the generation of cranial differences across the bovid tree. Rather, the distribution of bovid cranial morphospace appears to be mainly the result of constraints imposed by a deeply conserved size-shape allometry, and dietary diversification the result of adaptation of existing allometric pathways.

## Abstract (web summary)

Bibi and Tyler analyse the morphometric variation in the skulls of 96 species across Bovidae, using 3D geometric morphometrics and ecological variables. They show that ecological variables alone appear to have little connection to shape disparity in bovid skulls, instead discovering a deeply conserved association between size and shape that is linked to dietary selection.

## Introduction

Despite the importance of historical factors, structural constraints, or even contingency on the role of morphology^1^, we still intuitively favor adaptationist explanations for evolutionary differences among taxa. This is particularly true when phenotypic differences are large and appear to be correlated with some obvious changes in behavior or environment. Since the late 20th century, the study of evolutionary morphology has sought to tease apart the different influences that drive the ontogeny and evolution of shape, and technological and statistical developments have provided a continually expanding toolkit for comparisons of biological form. One such development is geometric morphometrics (GM), which has been applied to examine morphological development and rates of change across diverse parts of the tree of life^2,3^. The power of GM lies in its ability to quantify form in a continuous, multidimensional space, which permits examination of the roles of both extrinsic (climate, diet) and intrinsic (size, phylogeny) variables on the generation of shape.

**Fig. 1:**
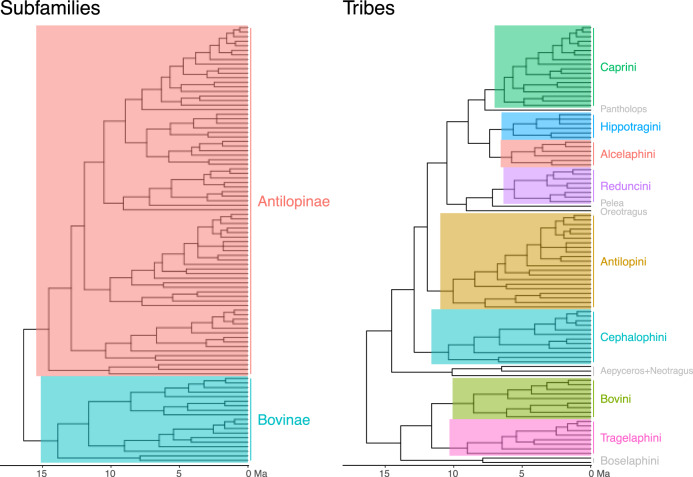
Dated phylogeny of Bovidae with species used in this study. Subfamilies and tribes with more than three species are highlighted. Tree and ages are based on Faurby and Svenning^51^.

With ~140 extant species^4^ spread across four continents, bovids (antelopes and relatives) comprise the most taxonomically diverse family of extant terrestrial large mammals. Divided into two subfamilies and ~13 tribes (Fig. 1), bovids today can be found in habitats ranging from tropical forests to arctic tundra, with individual species typically exhibiting well-defined climatic and dietary preferences. The affinities of most bovid species, genera, or tribes to particular climates or habitats means the relative abundance of bovids has been used extensively for paleoecological reconstruction, particularly in the African Plio-Pleistocene fossil record (e.g., refs. ^5–7^). However, while distinguishing the head of an arctic muskox from that of a tropical waterbuck might be straightforward, we still do not understand the processes that shaped cranial diversity among bovids. The fact that climate and diet often differ significantly among species or tribes places these two extrinsic variables as prime suspects in any consideration of differences in morphology. Several early studies found correlates of particular skull traits or metrics with such environmental variables (e.g., refs.  ^8,9^), but these approaches did not take phylogeny into account, so their results are difficult to interpret. More recent work that incorporated phylogeny found support for a relationship between dietary ecology and traits such as hypsodonty, facial elongation, and mandibular shape^10,11^. Other studies have examined bovid cranial shape using three-dimensional morphometrics but focused mainly on aspects of evolution and development (e.g., allometry, integration) without testing for environmental correlates^12,13^. Size has been repeatedly shown to be a strong predictor of shape in vertebrate crania and size-shape allometry provides a simple but powerful mechanism for the generation of morphological diversity without developmental novelty^12,14,15^, but we still do not know the role of size allometry in generating the range of bovid cranial shape observable today. In addition, anatomical modularity has often been implicated in the generation of phenotypic novelty as a result of the greater evolvability it confers^16–18^, but it is unclear if modularity played a role in generating extant bovid cranial disparity.

Here, we used three-dimensional GM to examine the shape of the bovid cranium and to investigate the environmental pressures and generative processes underlying it. We assessed the relationships between cranial shape, phylogeny (including subfamilies and tribes), three discrete environmental variables (diet, habitat, net primary productivity), and three continuous phenotypic variables (size, facial elongation, hypsodonty). Based on their strong habitat-specificity, their differentiation into a broad range of environments, and their high cranial morphological diversity, we expected to find strong correlations of cranial shape with environmental and dietary variables and a strong phylogenetic partitioning of morphospace. We tested the following hypotheses: cranial shape shows high phylogenetic dispersion and low levels of convergence among tribes (Hypothesis 1). Cranial shape is correlated with environmental variables, including diet and habitat (Hypothesis 2). Modularity played an important role in the generation of morphological differences among bovid lineages (Hypothesis 3).

## Results

### Landmark rarefaction

Rarefaction of the landmark dataset indicated that the number of landmarks used (*n* = 53) was sufficient for capturing the diversity of cranial shape that it covered, with some 90% of the shape variation in the parent dataset captured after just 30 landmarks, and almost 100% of centroid size variation captured by just 10 landmarks (Supplementary Fig. 1).

### Scan type

Differences in shape between scan types (Artec vs. CT) were nonsignificant (ols ANOVA, *P* = 0.90, *Z* = −1.25). In addition, there are no groupings of Artec or CT specimens together in the UPGMA phenogram of all specimens (Supplementary Fig. 2, see Supplementary Materials for which specimens were scanned with which method). Therefore, we do not expect differences in scan types to have affected our results.

### Intraspecific variation

The UPGMA phenogram of the Procrustes coordinates for all 141 specimens showed that, for the most part, specimens of the same species clustered together, indicating that intraspecific differences were mostly smaller than those between species (Supplementary Fig. 2). Intraspecific variation along the first two principal components (PCs) is also shown in Supplementary Fig. 3, providing a further sense of the variation introduced by different iterations of the analyses on the primary shape components. Numerous iterations of the random subsetting procedure produced almost identical results for most analyses below, indicating that intraspecific differences, while important to consider, did not significantly affect our conclusions.

### Principal components analysis of the phylogenetic subset

**Fig. 2:**
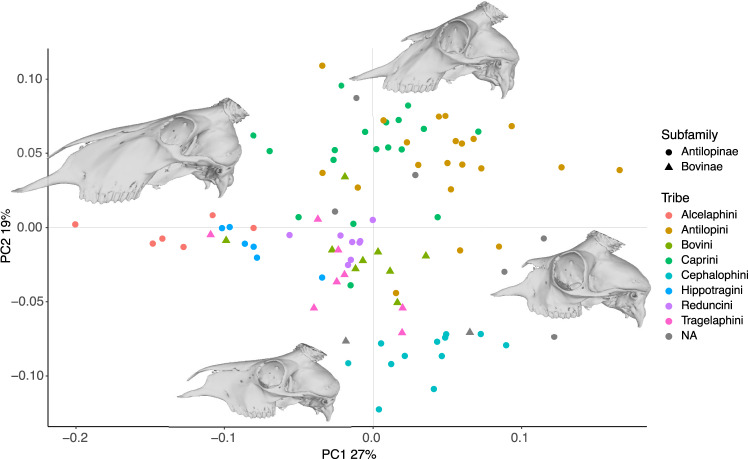
First two principal components of the analysis of 96 crania representing 96 species. In lateral view, PC1 captures mainly facial elongation and both PC1 and PC2 capture aspects of facial depth and nasal retraction. Reconstructions were made by warping a cranium of *Naemorhedus goral* (AMNH 43033) to extreme PC values. See Supplementary Fig. 4 for a version with species labels.

**Fig. 3:**
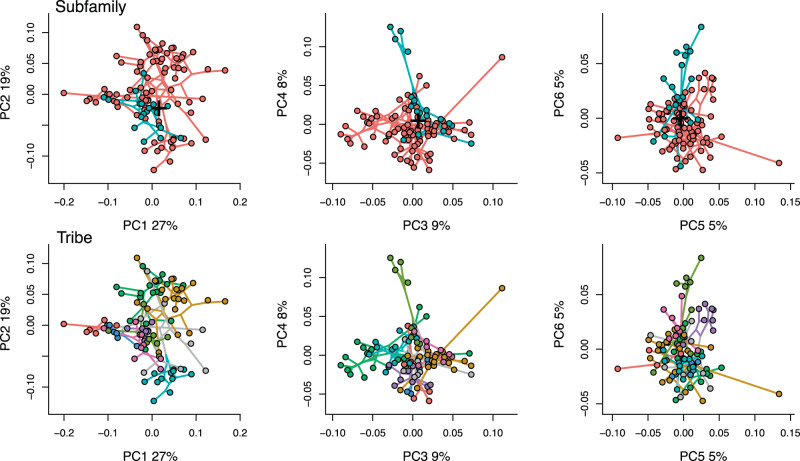
Phylomorphospace along the first six principal components. Whether examined by subfamily or tribe, bovid cranial morphospace shows high overlap and poor phylogenetic differentiation. The black + marks the reconstructed position of the most recent common ancestor of all extant bovids (root node in Fig. 1). The correspondence between colors and clades is the same as in Fig. 1.

The first three PCs explained 26, 18, and 9% of the variance, respectively, and the first 17 PCs explained 90% of the variance. Figure 2 shows the PC1-PC2 morphospace for the phylogenetic subset (Supplementary Fig. 4 shows the same with species labels). PC1 appears to reflect primarily facial elongation, facial deepening, and braincase flexion (negative is more), while PC2 also reflects facial elongation and deepening along with orbital protrusion (positive is more) (Fig. 2 and Supplementary Fig. 5). Correlates of PC1 are explored further below. Figure 3 shows bovid cranial phylomorphospace plotted along the first six PCs, showing high overlap among subfamilies and tribes, and placement of the bovid most recent common ancestor close to the mean shape. Supplementary Fig. 6 shows the distribution of species along the first 19 PCs, also showing consistently strong clustering and high overlap around the mean.

### Differences in shape by taxon

**Fig. 4:**
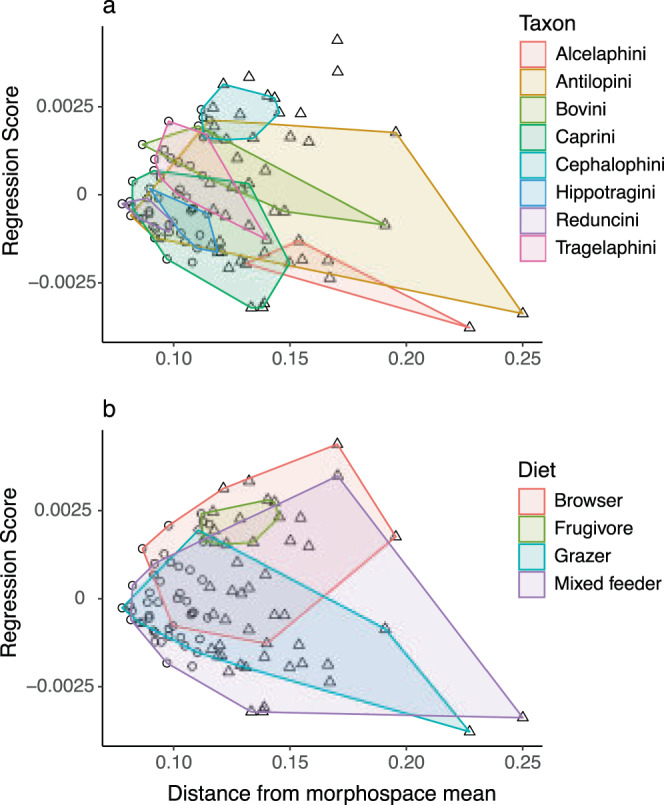
Increasing divergence in shape (regression score of shape on distance) with increasing distance from the morphospace mean. Convex hulls highlight (**a**) tribes, and (**b**) dietary categories. Phylogenetic and dietary differences in shape become more significant with increasing distance from the mean, or between peripheral (triangles) and central (circles) species. Note some individuals in (**a**) do not belong to any of the eight main tribes. See Supplementary Fig. 7 for a version with species labels.

**Fig. 5:**
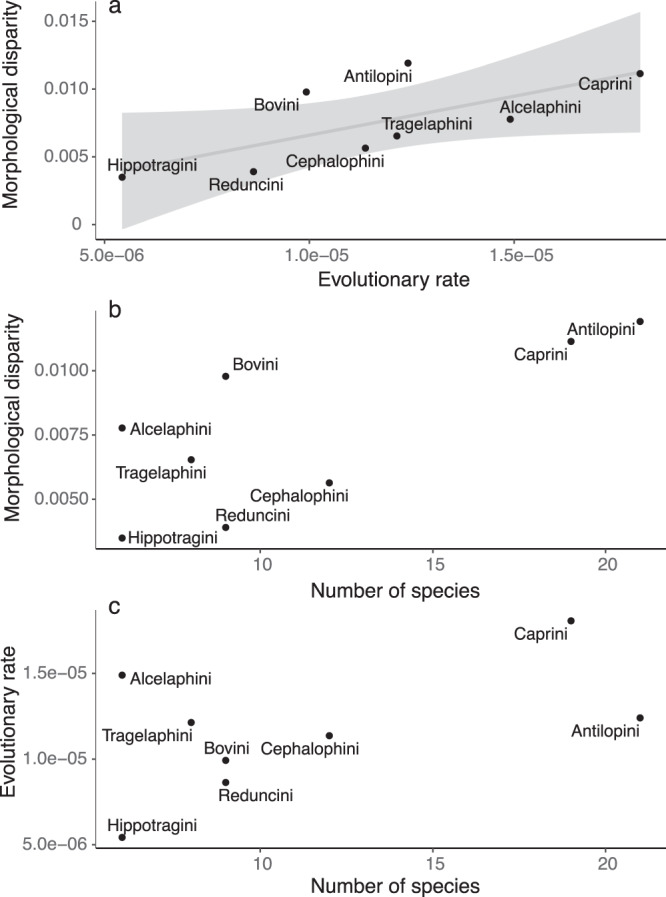
Morphological disparity in relation to evolutionary rates and species richness among tribes. **a** Disparity and rates of morphological change are strongly correlated (though the pairwise differences among tribes are largely nonsignificant, see the text). However, neither disparity nor rates are significantly correlated with clade richness (**b**, **c**), implying that cranial shape is not a major determinant of speciation in bovids.

Multivariate phylogenetic signal (Blomberg’s *K*) in cranial shape was 0.62 and significant (*P* = 0.001). For just Bovinae, *K* = 0.60 (*P* = 0.001), and for Antilopinae, *K* = 0.73 (*P* = 0.001). Among tribes, phylogenetic signal was significant and ranged between ~0.6 and 1.1 (*P* < 0.05) for all tribes except Cephalophini and Tragelaphini, in which phylogenetic signal was nonsignificant (*K* = 0.44, *P* = 0.18 and *K* = 0.51, *P* = 0.32, respectively). Cranial shape evolution in bovids, therefore, can generally be described as Brownian (*K* ~ 1) but with a high degree of homoplasy or stasis (*K* < 1). Shape differences between subfamilies and tribes (Fig. 3) were highly significant (ols ANOVA, shape~subfamily, shape~tribe, Table 1). Pairwise tests among tribes indicated significant shape differences among all comparisons (*P* < 0.05, *Z* > 1.7) except Alcelaphini to Hippotragini (*P* = 0.21, *Z* = 0.9). Taking into account the distance of species from the morphospace mean (Fig. 4a) resulted in a further improvement of fit between shape and tribes (ols ANOVA, shape ~ tribe * distance, interaction of tribe and distance *P* = 0.002, *Z* = 3.3). This indicates that differences in shape among tribes are more pronounced toward the extremes of morphospace, further confirming the strong relationship between phylogenetic and morphological divergence (Fig. 4a). Differences in morphological disparity (Procrustes variance) and evolutionary rates between subfamilies were both nonsignificant (*P* = 0.17, and *P* = 0.71, *Z* = −0.65, respectively). This indicates that the greater taxonomic diversity in Antilopinae is not associated with a significantly broader range of morphology, and is also not the result of increased rates of morphological innovation in cranial shape. Among tribes, Antilopini, Caprini, and Bovini exhibited significantly higher disparity than Cephalophini, Hippotragini, and Reduncini (*P* < 0.05, Fig. 5), but other pairwise comparisons were nonsignificant. Rate differences among tribes were significant (*P* = 0.008, *Z* = 3.31), though pairwise tests indicated this was almost entirely due to slow rates in Hippotragini, and almost all other pairwise comparisons were nonsignificant. Relationships of species richness to disparity and evolutionary rates were nonsignificant among subfamilies and tribes (*P* = 0.09, *Z* = 1.2 and *P* = 0.22, *Z* = 0.73, respectively, Fig. 5). In other words, taxonomic and morphological diversification, at least as far as the cranium is concerned, were largely decoupled.

**Table 1:** Relationship of cranial shape and log-centroid size to the most important variables examined in this study (individual ANOVAs using residual randomization).

	Df	R^2^	F	Z	P	Type
Shape~						
Tribe * Distance from mean	14	0.57	6.52	11.13	0.001	ols
Tribe	7	0.47	10.16	10.86	0.001	ols
Size + Facial length	2	0.21	12.23	7.07	0.001	pgls
Facial length	1	0.15	17.03	6.03	0.001	pgls
Size * Diet	7	0.17	2.55	5.86	0.001	pgls
Size	1	0.09	9.54	5.72	0.001	pgls
Diet * Distance from mean	7	0.13	1.95	4.10	0.001	pgls
Subfamily	1	0.05	4.91	3.47	0.001	ols
Hypsodonty	1	0.04	2.27	2.29	0.01	pgls
Diet	3	0.04	1.28	1.27	0.11	pgls
Habitat	4	0.10	1.38	1.19	0.11	ols
NPP	3	0.04	0.79	−0.47	0.68	ols
Size~						
Tribe	7	0.68	24.3	8.76	0.001	ols
Facial length	1	0.42	67.09	4.79	0.001	pgls
Subfamily	1	0.27	34.98	4.03	0.001	ols
Diet	3	0.15	5.30	2.97	0.003	pgls
Hypsodonty	1	0.16	10.56	2.55	0.002	pgls
Habitat	4	0.05	0.72	−0.20	0.58	ols
NPP	3	0.00	0.03	−2.47	0.996	ols

After phylogeny (represented by subfamilial and tribal differences), facial elongation and size provide the strongest explanations of shape, while diet, habitat, and net primary productivity (NPP) are not significant predictors of shape. Size explains large proportions of the variation in facial length, diet, and hypsodonty. Differences among subfamilies and tribes are of borderline significance, and differences among habitats and NPP categories are not. All tests with interaction factors (*) have significant interaction effects (results given in the text). pgls (phylogenetic least squares) tests take phylogeny into account while ols (ordinary least squares) tests do not. Tests with tribe, hypsodonty, habitat, and NPP use reduced species subsets. Results sorted by *Z* score (effect size).

### Diet

The relationship of diet to total shape was nonsignificant (Table 1). The fit of diet to shape improved slightly when a lower number of shape dimensions was considered (e.g., the first 17 PCs, *P* = 0.05, *Z* = 1.75). Pairwise comparisons (using total shape) indicated browsers can be distinguished from mixed feeders and grazers with borderline significance (*P* = 0.05, *Z* = 1.7 for both), and all other pairwise comparisons were nonsignificant. However, the interaction of diet with distance from the morphospace mean was highly significant (pgls ANOVA, shape ~ diet * distance, interaction effect *P* = 0.001, *Z* = 4.1) and improved the fit of diet to cranial shape considerably (Table 1 and Fig. 4b). Pairwise comparisons of slopes on this fit were all significant (*P* < 0.05, *Z* > 2.0), except the comparison of browsers to frugivores (*P* = 0.19, *Z* = 0.9). Considering central vs. peripheral species separately (Fig. 4, labeled version as Supplementary Fig. 7), shape and diet were of borderline significance among central species (pgls ANOVA, shape ~ diet, *P* = 0.06, *Z* = 1.6), with pairwise comparisons of means distinguishing browsing from mixed-feeding taxa (*P* = 0.006, *Z* = 2.4), but significantly different among peripheral species (*P* = 0.01, *Z* = 2.3), distinguishing browsers from grazers and mixed feeders (*P* = 0.003, *Z* = 2.98, and *P* = 0.02, *Z* = 2.15, respectively). These results suggest that diet may play a role in the expansion of morphospace toward the peripheries (i.e., in increasing disparity).

### Habitat & net primary productivity

**Fig. 6:**
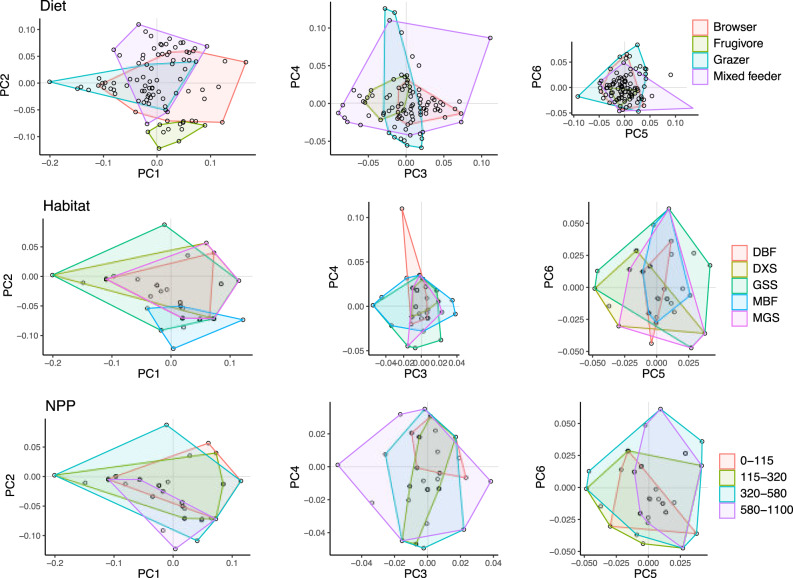
Distribution of diet, habitat, and net primary productivity (NPP) categories in shape space. Habitat and NPP use just 20% most commonly occurring species in each category (see also Supplementary Fig. 8). Some discrimination can be seen along PC1 and PC2, but otherwise all categories exhibit high morphological overlap and differences are not significant. Habitat abbreviations: DBF tropical and subtropical dry broadleaf forests, DXS tropical and subtropical deserts and xeric shrublands, GSS grasslands, savannas, and shrublands, MBF moist broadleaf forests, MGS montane grasslands and shrublands. NPP is in trillions of kgs of carbon per 1 degree square.

Habitats showed high overlap in cranial shape, even when considering just the 20% most common species in each habitat type (Fig. 6, and see Supplementary Fig. 8 for maps of habitat distributions and results using the 50% most common species). Differences in shape among the five habitat types were nonsignificant (ols ANOVA, shape ~ habitat, Table 1). Pairwise tests indicate that the only consistent significant shape differences were between the most extreme habitats: moist broadleaf forests (MBF) and deserts and xeric shrublands (DXS) (*P* < 0.05, *Z* = 2.6). This single pairwise comparison remained significant even when using the set of 50% most common species or all species, but the use of phylogenetic tests could reduce significance as these habitats include large numbers of closely related species (e.g., Cephalophini in MBF). Similarly, NPP categories showed high overlap in cranial shape, even when considering just the 20% most common species in each category (Fig. 6 and Supplementary Fig. 8). Differences in shape among NPP categories were not significant (ols ANOVA, shape~NPP, Table 1), and pairwise tests did not distinguish any comparisons of NPP bins.

### Hypsodonty

**Fig. 7:**
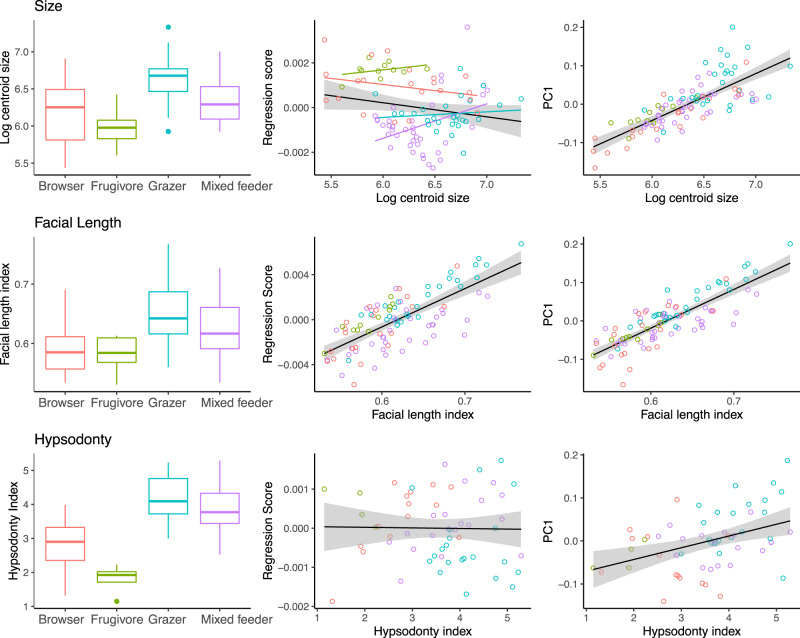
Relationships of size, hypsodonty, and facial elongation to diet and shape (regression score of shape against each variable). All three traits are significantly correlated with dietary differences as well as with total shape and PC1. These traits explain dietary differences better than total shape, and size and facial length together account for over 80% of the variance in PC1 (see the text). Differences in size-shape allometry (slopes) among dietary categories are significant, with pairwise tests distinguishing frugivores (i.e., Cephalophini) from all other categories and browsers from mixed feeders.

Hypsodonty exhibited significant phylogenetic signal and approximated evolution by Brownian motion (*K* = 0.84, *P* = 0.001). Antilopinae had significantly higher mean hypsodonty than Bovinae (ols, hypsodonty ~ subfamily, *P* = 0.01, *Z* = 2.12, Supplementary Fig. 9). Differences among tribes were also significant (*P* = 0.001, *Z* = 5.13), and pairwise tests showed these mostly to be among comparisons of Alcelaphini (the most hypsodont tribe) and Cephalophini and Tragelaphini (the least hypsodont tribes) against remaining tribes (ols ANOVA, hypsodonty ~ tribe, *P* < 0.05, *Z* > 1.8). Hypsodonty was strongly correlated with cranial shape, but explained only 4% of the variance and had a slope close to zero (pgls ANOVA, shape~hypsodonty, Table 1 and Fig. 7). However, hypsodonty was strongly correlated with size, with more hypsodont species tending to be larger (pgls ANOVA, size~hypsodonty, Table 1), as well as with PC1 (pgls ANOVA, PC1~hypsodonty, *P* = 0.001, *Z* = 2.87, *R*^2^ = 0.20), and with diet (pgls ANOVA, hypsodonty~diet, *P* = 0.001, *Z* = 6.01, *R*^2^ = 0.55).

### Facial length

Facial length exhibited a significant but low phylogenetic signal (*P* = 0.001, *K* = 0.55), indicating a high degree of homoplasy. Differences in facial length between subfamilies were of borderline significance (ols ANOVA, facial length ~ subfamily, *P* = 0.06, *Z* = 1.55), while those among tribes were significant (ols ANOVA, facial length ~ tribe, *P* = 0.001, *Z* = 7.6), with the major differences among comparisons of Alcelaphini, Hippotragini, and Caprini (long faces) against Antilopini, Cephalophini, and Tragelaphini (short faces, Supplementary Fig. S9). Facial length was significantly correlated with cranial shape (pgls ANOVA, shape ~ facial length, Table 1) and with dietary differences (pgls ANOVA, facial length ~ diet, *P* = 0.002, *Z* = 2.92), with the main pairwise differences between grazers and browsers, and grazers and frugivores (both *P* < 0.05, *Z* = 2.4). Facial length was also significantly correlated with size (pgls ANOVA, facial length ~ size, Table 1), and with PC1 (pgls ANOVA, facial length~PC1, *P* = 0.001, *Z* = 7.05, *R*^2^ = 0.74).

### Size

Log-centroid size was highly correlated with species average body mass as derived from the literature (58 species, *P* = 0.001, *R*^2^ = 0.88, Supplementary Fig. 10). Centroid size exhibited significant phylogenetic signal and approximated evolution by Brownian motion (*K* = 1.13, *P* = 0.001). Differences in size between subfamilies and among tribes were significant (ols ANOVA, size~subfamily, size~tribe, Table 1 and Supplementary Fig. 9). Pairwise tests of means were significant for comparisons of Antilopini and Cephalophini (the smallest tribes) against all other tribes (*P* < 0.05, *Z* > 1.9). Size was significantly different among dietary categories (pgls ANOVA, size ~ diet, Table 1 and Fig. 7), with the main pairwise differences in comparisons of grazers against mixed feeders and browsers (*P* < 0.05, *Z* > 1.9). Size was significantly correlated with cranial shape, and explained almost 10% of the variance (Table 1 and Fig. 7). Differences in size-shape allometry between subfamilies were nonsignificant (pgls, shape~size * subfamily, interaction effect *P* = 0.20, *Z* = 0.85, test of slopes *P* = 0.18, *Z* = 0.95), as were differences among tribes (shape ~ size * tribe, interaction effect *P* = 0.13, *Z* = 1.15). Pairwise comparisons of slopes (Supplementary Fig. 11) indicated significant differences between Cephalophini and some other tribes (Bovini, Tragelaphini, Caprini, *P* < 0.05, *Z* > 1.9), though small sample sizes may be an issue here^19^. These results indicate that bovids share broadly similar allometric relationships. Differences in size-shape allometry among dietary categories were, however, significant (pgls, shape~size * diet, interaction effect *P* = 0.02, *Z* = 2.2), though sample sizes were also small. Pairwise tests of slopes distinguished frugivores (i.e., Cephalophini) from all other categories and browsers from mixed feeders (*P* < 0.05, *Z* > 1.8). Size was highly correlated with PC1, accounting for ~60% of the variance (pgls ANOVA, PC1~size, *P* = 0.001, *Z* = 6.47, *R*^2^ = 0.63), and size and facial length together explained over 80% of the variance (pgls ANOVA, PC1~size + facial length, *P* = 0.001, *Z* = 11.21, *R*^2^ = 0.83). Size was significantly correlated with both hypsodonty and facial length, but not with habitat type or NPP category (Table 1).

### Modularity

Cluster analyses of Procrustes shape coordinates along with the gap statistic using the two different covariance matrix approaches produced slightly differing results. Using the method of Lucas and Goswami^20^ resulted in the recognition of two modules largely distinguishing the orbital area from the remainder of the cranium (Supplementary Fig. 12). Using the phylogenetically informed covariance matrix and distance method of Griffin^21^, the gap statistic indicated the presence of between three and five modules depending on the iteration of the analysis. However, all three of these solutions identified modules comprised of just one or two coordinates, which was not biologically meaningful. We therefore chose to conservatively recognize just two modules, distinguishing mainly parts of the dorsal cranium from the remainder of the cranium (Supplementary Fig. 12). The identification of the orbital region as a module in the first scheme and the dorsal cranium midline in the second plausibly reflects high interspecific variation in the orientation and length of the orbits and nasals, in the size and the shape of the frontal perhaps as a function of horn core basal size and location (and possibly sexual dimorphism), and of flexion of the angle between the braincase and face. Examining these two modularity schemes along with the five predefined schemes using maximum likelihood model selection using EMMLi found the nine-module scheme (scheme 4) with separate true correlation coefficient (rho) values within and among each module to be the most likely. However, this was also the most parametrized scheme, and it appears that EMMLi’s likelihood scores are highly correlated with the number of model parameters (Pearson’s correlation coefficient = 0.78, *P* < 0.001, Supplementary Fig. 13). Felice & Goswami^22^ also found the most parameterized modularity schemes they tested to be the most likely, but then took the additional step of combining modules that had high between-module correlation values (rho > ~ 0.5). The highest between-module rho value in our nine-module scheme was 0.37, and most values were below 0.25, which did not provide a basis for the pooling of modules. The possibility of finding even more likely models by proposing even more modular schemes sheds some doubt on the usefulness of this approach for objectively identifying modularity.

Phylogenetic null-model tests of the seven predefined modularity schemes show that the first five schemes all show significant modular signal, with the nine-module “Element” scheme having the lowest covariance ratio (CR) and most negative effect size (*Z*), which indicates the strongest modular signal (Table 2). As with EMMLi, CR values favored the most parametrized scheme, but here the relationship between the number of modules and *Z* score was not significant (Pearson’s correlation coefficient = −0.61, *P* = 0.14, though the sample size was small, *n* = 5). Tests for differences in levels of modularity between subfamilies and among tribes using the nine-module scheme indicated no significant differences (*P* = 0.41, *Z* = 0.81 and *P* > 0.1, *Z* < 1.6 for subfamilies and tribes, respectively).

**Table 2:** Results of phylogenetic modularity tests using the covariance ratio (CR).

Scheme	*n* Modules	CR	*Z*	*P*
Element	9	0.69	−3.35	0.001
Tripartite	3	0.79	−2.91	0.002
Mammalia 6	6	0.7	−2.88	0.003
Mammalia 2	2	0.84	−2.83	0.005
Four	4	0.81	−2.68	0.005
Clustering 2	2	0.94	−0.22	0.407
Clustering 1	2	0.95	0.06	0.509

Five of the seven schemes are significantly different from a phylogenetic random null model, but the most parametrized model (Element) has the strongest modular signal (most negative *Z* score). The two models identified on the basis of the clustering of coordinate covariance matrices (Clustering 1 and 2) do not show significant modular signal.

## Discussion

We did not find support for any of our three working hypotheses. Instead of being widely dispersed (Hypothesis 1), bovid cranial morphospace is highly clumped (Figs. 2 and 3 and Supplementary Fig. 6). Even though subfamilies and tribes can be distinguished in shape (Table 1), overlap is high, and species belonging to tribes as different as Alcelaphini (grazers in grasslands), Tragelaphini (browsers in wooded to forest habitats), and numerous Bovini and Caprini, (including diverse diets from a broad range of habitats) share broadly similar cranial shapes (Fig. 3 and Supplementary Fig. 2). In addition, while phylogenetic signal is present in cranial shape, it is low, indicating higher degrees of homoplasy (convergence) than expected under a Brownian motion model of evolution. For two tribes, Tragelaphini and Cephalophini, phylogenetic signal in cranial shape is even nonsignificant. These results reflect the high rate of homoplasy typical of bovids, which has been a major challenge to all previous attempts to construct stable phylogenies (or for that matter, taxonomies) based on morphological analyses^23,24^. Furthermore, the finding that different bovid subfamilies and tribes do not differ significantly in their rates of morphological evolution or morphological disparity, despite differing greatly in their species diversity, indicates a decoupling of taxonomic and morphological diversification that might also have been hindering historical attempts to adequately resolve phylogenetic relationships. However, we only considered overall cranial shape, and, since bovid species are often diagnosed on the basis of horn morphology or skin patterning, further studies may want to consider these features in relation to diversity and disparity. The radiation of Cephalophini across African forest habitats has, for example, resulted in the recognition of numerous species largely differentiated by size and pelage, but when cranial shape is considered alone, this radiation exhibits low disparity, high clustering, and no phylogenetic signal.

Furthermore, we found that the relationships between cranial shape and diet, habitat, and net primary productivity were largely nonsignificant (Hypothesis 2, Table 1). Nonetheless, browsers could be distinguished from mixed feeders and grazers, and differences in cranial shape between bovids inhabiting the most extreme habitats (DXF vs MBF) were significant. While diet did not show a significant correlation with cranial shape, the interaction between diet and the distance of a species from the morphospace average did, which tentatively suggests some role for diet in driving morphospace expansion to the extremes. In contrast to total shape, dietary differences were found to be strongly correlated with all three phenotypic traits considered (hypsodonty, facial elongation, and size), indicating a much greater influence of diet (and probably correlated climatic factors) on the evolution of individual phenotypic traits than on total cranial shape. This explains how some bovid clades could share similar cranial form but still be strongly distinguished ecologically: changes in individual traits (e.g., hypsodonty), are probably more critical to dietary adaptation than changes in overall cranial shape.

In fact, our most important finding may be the strong relationship that a single trait—size—has on cranial shape, dietary differences, and traits such as hypsodonty and facial elongation (Fig. 7 and Tables 1 and 2). Size exerts a strong influence on the cranial shape and explains most of the variance along its first principal component, which separates small, short-faced antelopes such as dik-diks (*Madoqua* spp.) from large, long-faced alcelaphins (e.g., *Alcelaphus buselaphus*). The association of longer faces with larger size is thought to be a deeply conserved relationship across placental mammals (positive craniofacial allometry)^12,15^ and bovids appear to be strongly bound to this ancestral constraint. Over macroevolutionary timescales, the constraints of size allometry have probably limited the generation of novel shape in bovid crania to a few dimensions^25,26^. At the same time, size is also strongly correlated with diet, and, while diet itself is not significantly correlated with cranial shape, the interaction of size and diet is significant and significantly improves the proportion of total shape explained by size (Table 1). We also found that size-shape allometries among species adapted to different dietary strategies are also significantly different, in particular for the frugivorous Cephalophini (Fig. 7). The relationship of size, shape, and diet therefore appears to have provided a simple but powerful method for the generation of morphological novelty in bovid crania. A similar pattern has been documented in numerous other clades, including bats, rodents, and monkeys, with dietary adaptations associated with size and shape changes via existing shape allometry, termed a “line of least evolutionary resistance”^14,27,28^.

As noted above, there is a strong relationship between facial lengthening and size as predicted by positive craniofacrial allometry^15^, but we also found that facial elongation in bovids differs significantly among dietary categories and that facial length and size together explain almost twice the variance in shape explained by size alone (Table 1). The relationship of body size to diet among herbivores has been well established (the Jarman–Bell principle)^29^, and it is therefore not a coincidence that large bovids are often grazers, or that the smallest bovids are mostly browsers or frugivores. Similarly, previous work that found a relationship between facial lengthening and grazing diets in bovids^10^, and references therein, e.g., ref. ^30^, possibly related to increased size of the masseter muscles for enhanced grinding^31^. So our findings suggest that dietary selection may have played a role in further enhancing aspects of shape initially generated by the conserved size-shape allometric relationship. Since size might be expected to evolve much faster than changes in skull shape, this may be an example of exaptation^32^ whereby the allometric effects of rapid changes in size are exapted for new functions. The bovid fossil record should be examined for evidence of changes in size preceding changes in shape.

Bovid cranial shape is undoubtedly the result of a complex combination of factors, and, beyond the dominant effects of constraints such as size-shape allometry, further adaptive explanations such as sexual selection, locomotion, or species-specific physiological needs are worth further investigation. Sexual dimorphism in bovids is mostly expressed in larger body size and the possession of horns in males, or in the larger size of the horns in species with horned females. Because of their importance for sexual signaling, male–male competition, and presumably species recognition, species-level morphological differences are most apparent in the horns ^33^. These differences naturally affect the size and shape of the dorsal cranium and may be a prominent influence on total cranial shape. In addition, while all bovids are terrestrial and cursorial, differences in locomotory needs based on foraging or predator evasion behaviors might also have played a role in shaping (or limiting) cranial morphospace. Finally, species-specific physiological needs might have shaped other conspicuous cranial features. Nasal retraction as observed in *Madoqua* and *Saiga* species, for example, appears to figure strongly in the extremes of PC1-PC2 (Fig. 2 and Supplementary Fig. 5). The function of the fleshy protruding noses in these species is yet uncertain, but dust-filtering and vocalization functions have been proposed^34,35^.

Historical contingency might also be considered, and in the case of bovid cranial form, while the distribution of certain traits (e.g., size, facial length) do not differ from expectations of a phylogenetic random walk, cranial shape overall shows lower levels of divergence than predicted by phylogenetic inertia alone (ie. low values of Blomberg’s *K*). This, in conjunction with conserved craniofacial allometry, possibly implies a role for stabilizing selection in maintaining an average bovid cranial shape, which itself may have provided a good template for ecological generalization across bovid tribes. Ancestral state reconstruction suggests that the early Miocene most recent common ancestors of all bovids, as well as the ancestors of subfamilies Bovinae and Antilopinae, exhibited mean morphological cranial shape (Fig. 3), but this should be confirmed through further work, including on fossil species. Such a long persistence of the family’s grand mean might suggest the bovid cranium may be an example of what Gould^36^ termed an “actively stabilized bauplan”. While Gould preferred intrisic (e.g., developmental) reasons behind the stabilization, the phenomenon more likely arises from a combination of both intrinsic (e.g., conserved craniofacial allometry) and extrinsic (stabilizing selection) factors (e.g., refs. ^14,28^).

Our last hypothesis (Hypothesis 3) is also not supported, as we find no consistent evidence for modularity having played a role in the generation of morphological differences across the bovid tree. Despite numerous studies claiming the importance of modularity in the evolution of cranial disparity, the search for modularity in high-dimensional data has recently come under fire, and it appears that neither high likelihood values nor low CR scores provide conclusive evidence for the presence of modularity in the evolution of shape^37,38^. The fact that most modularity schemes we tested were statistically significant (Table 2) should be taken with caution, and we suspect this might simply reflect the spatial autocorrelation of morphology across an otherwise highly integrated structure. This leaves open the possibility that different regions of the cranium could evolve at different rates (i.e., mosaic evolution), as found in numerous studies that studied modularity^22^, but without necessarily conforming to well-defined anatomical modules. More importantly for our purposes, even using the most modular scheme, we found no differences in levels of cranial modularity among subfamilies and tribes, indicating no major role for modularity in the generation of phenotypic novelty among bovid clades.

Bovids therefore appear to be an example of a highly adaptable clade with low evolvability. Despite their diversification into a wide range of terrestrial environments, bovids occupy a fairly conserved adaptive landscape, at least as far as cranial shape (excluding horn and dental morphology) is concerned. While the relationship between diet and individual cranial traits (size, hypsodonty, facial elongation) is strong, the influence of diet is largely watered down when the total cranial shape is considered, probably indicating a complex array of factors at play, including convergence and probably stabilizing selection. Further adaptive factors could be explored such as sexual selection or species-specific adaptations, but what is clear is that intrinsic factors play a fundamental role in limiting the range of shape variation seen across bovid crania. Much of this appears to be the result of a deeply conserved size-shape allometry common to all placental mammals^15^. Rather than requiring novel structures, the functional needs of dietary adaptation in bovids appear to have been met through modifications of existing size-shape trajectories, or through exaptation of the morphological effects of body size changes. These findings are in agreement with studies arguing for a primary role for the evolvability of size in the generation of morphological disparity.

## Methods

### Statistics and reproducibility

All analyses were conducted in R v.4.0^39^ and R Studio v.1.3^40^ and the majority of analyses relied on the *geomorph* v.4.0 and *RRPP* v.1.0 packages^41–43^.

### Specimen digitization and landmarking

**Fig. 8:**
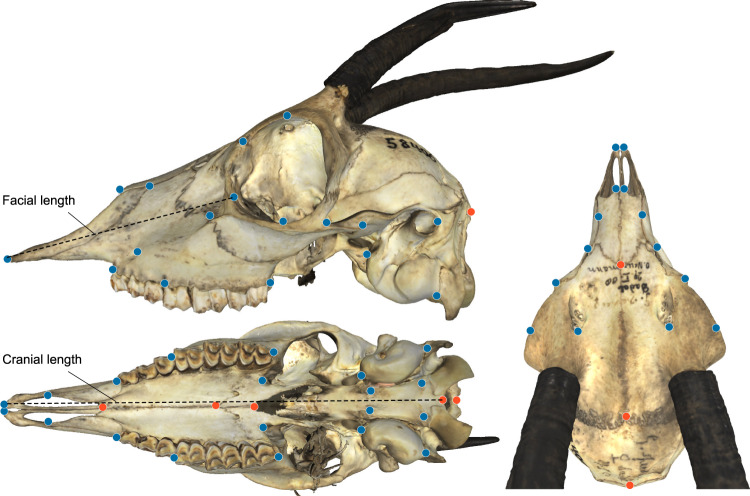
Landmarks used in this study, The 53 cranial landmarks are shown on a 3D surface model of a female *Gazella spekei* (ZMB Mam 58406). Bilateral landmarks in blue, midline in red. Shown also are the two distances measured to calculate the facial length index.

3D surface models of 141 bovid crania from the collections of the Museum für Naturkunde, Berlin, American Museum of Natural History (AMNH), and Yale Peabody Museum (YPM) were generated using the Artec Spider or Eva surface scanner or from CT scans (YPM and AMNH specimens used scans from Farke^44^). In total, these represent 96 species following the IUCN taxonomy at iucnredlist.org (which currently recognizes 139 bovid species). All are extant, with the exception of *Bos primigenius*, which is technically extinct though its lineage survives in domesticated cattle. To minimize the effects of sexual dimorphism or ontogeny, specimen selection focused on males (where known), and adults. For five species (details in supplementary material), sufficiently preserved male crania were not available and females (or presumed females) were used. This is a small number, however, and we do not expect morphological differences due to sexual dimorphism to have had a significant effect on the total dataset. 53 landmarks chosen to capture total cranial shape excluding horns. These were digitized on each skull using iDAV Landmark Editor v3.6^45^ (Fig. 8). The large majority of landmarks identify discrete anatomical structures or sutural points that are easily replicable among multiple users (both authors conducted landmarking). However, two landmarks located on the dorsal and ventral orbital rim (10–11), and two on the temporal process of the zygomatic (14–15) were more difficult to objectively assign and are expected to have higher variation on account of this measurement error than other landmarks (with potential implications for the modularity analyses below). Missing landmarks were estimated using the thin-plate spline method. Landmark coordinates were transformed into Procrustes-aligned shape coordinates prior to all analyses.

### Specimen subsets

A few analyses used all 141 specimens, but the large majority of analyses used a subset with just one cranium per species (“phylogenetic subset”, 96 crania), made by randomly selecting a single specimen for those species represented by multiple specimens. This means each iteration of our analysis generated a sample with the same species, but a slightly different combination of individuals. The advantage of using random subsetting rather than species means is that it allowed us to incorporate some of the effects of intraspecific variation on our analyses. Exact statistical test results therefore varied slightly among iterations, and a range is given when these variations were large. Additional analytical subsets were made for tribe-level analyses, which included only bovid tribes with more than three species (eight tribes with 88 species, colored in Fig. 1), and for species for which hypsodonty data was available (*n* = 56, see below).

### Landmark rarefaction

In order to assess whether the number of landmarks adequately capture shape variation in this dataset, sampling curves for shape and centroid size variation were generated using the *lasec* function from the *LaMBDA* v.0.1.1 package^46,47^. Adequate sampling is represented by plateaus in the sampling curves.

### Intraspecific variation

A hierarchical cluster analysis on a Euclidean distance matrix of the Procrustes shape coordinates of all specimens was used to assess whether specimens of the same species clustered together or whether intraspecific differences in shape could in some cases be larger than differences among species. The effects of intraspecific variation were also visualized along the first two principal components of form.

### Principal components analysis

In order to examine the major components of form, and in order to visualize the distribution of species in low-dimensional cranial morphospace, a principal components analysis was performed on the Procrustes shape coordinates using the full dataset of 128 crania as well as on the phylogenetic subset of 96 crania.

### Method of 3D model acquisition

Since different scanning devices can introduce a bias^48^, shape differences between specimens generated using different scan types (Artec vs. CT) were assessed using ordinary least squares ANOVA. Only specimens belonging to species scanned with both scan types were included (*n* = 50).

### Phylogenetic and taxonomic differences

In order to examine the effects of phylogenetic inertia on cranial shape, phylogenetic signal in the Procrustes shape coordinates was measured using the multivariate version of Blomberg’s *K*^49,50^, for which values close to zero indicate weak or no phylogenetic signal (close relatives less similar than expected by chance), values around 1 are consistent with conserved signal and morphological evolution through Brownian motion, and values greater than 1 indicate strong phylogenetic divergence (i.e., close relatives are more similar than expected by chance). All analyses that took phylogeny into the account used a pruned version of the mammal tree of Faurby and Svenning^51,52^, which is based on genetic data. In order to compare morphospace occupation between subfamilies and among tribes, differences in shape were examined using ordinary least squares (ols) ANOVA with random permutation. Disparity and rates of evolution of shape were examined using phylogenetic generalized least squares (pgls) ANOVA with random permutation. Disparity was calculated as the Procrustes variance. Rates of shape evolution were calculated from the Procrustes shape coordinates along a phylogeny and under a Brownian motion model.

### Diet

Species were assigned to one of four dietary categories (browser, grazer, mixed feeder, frugivore) based on the literature^30^ (e.g., ref. ^53^). Differences in shape among dietary categories were tested using phylogenetic ANOVA with random permutation. In order to examine whether dietary pressures may have played a role in the expansion of cranial morphospace from the center to the peripheries, we tested for a significant interaction of diet with the Euclidean distance of each species from the morphospace mean. Additional comparisons were done by equally assigning species to central or peripheral categories based on their distance from the morphospace mean and testing for differences in the relationships of diet and shape.

### Habitat and net primary productivity (NPP)

Faunal lists were assembled of all bovid species that occur within a particular habitat or NPP categories. Habitat assignments used WWF Major Habitat Types^54^ downloaded from the Nature Conservancy Terrestrial Ecoregions dataset http://maps.tnc.org/gis_data.html. Only habitats with >40 1 × 1° global occurrences with more than three bovid species each were used. These were deserts and xeric shrublands (DXS), montane grasslands and shrublands (MGS), tropical and subtropical dry broadleaf forests (DBF), tropical and subtropical moist broadleaf forests (MBF), tropical and subtropical grasslands, savannas and shrublands (GSS). Geographic occurrences of bovids were downloaded from the IUCN Red List website^4^. These were extracted for 1 × 1° grids, keeping only those grids that had four or more bovid species, and only occurrences from Africa and Eurasia (*n* = 1995). Faunal lists for each habitat type were then generated by compiling occurrences from all grids of a particular habitat type using all species, as well as using just the top 50 and 20% most commonly occurring species. NPP was extracted from the SEDAC Human Appropriation of Net Primary Productivity (HANPP) Collection (November 2006)^55^. Mean NPP was calculated for all 1 × 1° grids with more than three bovid species (*n* = 2222). The total range of NPP was divided into four categories with equal numbers of observations as follows: 0–115, 115–320, 320–580, 580–1100, with units in trillions of kilograms of carbon per 1 × 1° grid. As for habitats, all, 50%, and the 20% most common species in each NPP category were examined. Regressions of shape on habitat and NPP categories used ols ANOVA (i.e. not taking phylogeny into account) because the same species frequently occurred in more than one habitat or NPP category.

### Phenotypic traits

Three phenotypic traits (size, hypsodonty, facial length) were selected for comparisons with both cranial shape and environmental factors. Size was calculated as log-centroid size using the Procrustes shape coordinates and was compared with average body mass for 58 species by Mendoza et al.^30^ in order to examine its fidelity to actual body mass. Hypsodonty (lower m3 crown height divided by crown width) was taken from Janis^56^ and Mendoza et al.^30^ and was only available for the same subset of 58 species. Facial length was defined as the length of the face from the orbit to the anterior premaxilla divided by ventral cranial length and was measured using interlandmark distances (5–13 and 5–52, respectively) on the raw (nontransformed) coordinates. All tests of phenotypic traits against shape and dietary variables used pgls ANOVA with random permutation.

### Modularity

In order to assess the role that anatomical modularity might have played in the evolution of bovid cranial disparity, we tested for differing levels of modularity between subfamilies and across tribes. Prior to doing so, we compared multiple modularity schemes to identify that with the most modular signal to use for testing differences across the tree. Such assessments of modularity, which are currently quite popular in the GM literature, have been reported to be problematic, especially when using high-dimensional datasets with large numbers of 3D landmarks^37,38^. In addition, different methods, often with incomparable results, have been developed to test for or assess the presence of modularity and it is not clear which approach—if any at all—is the most appropriate. In an exploratory vein, we here applied several approaches to the generation and assessment of modularity schemes for the bovid cranium. First, five modularity schemes were defined as follows: (1) “Mammalia 2”: a two-module scheme that assigned coordinates to facial and neurocranial modules. (2) “Tripartite”: braincase, orbital, and anterior facial cranium. (3) “Four”: fronto-orbital, oral, snout, and temporal–occipital. (4) “Element”: nine modules: basioccipital, frontal, maxilla, nasal, occipital, orbit, palatine, premaxilla, temporal. (5) “Mammalia 6”: anterior oral-nasal, basicranium, cranial vault, molar, orbit, zygomatic-pterygoid. The first three schemes were based on Sanger et al.^57^ and Cardini & Polly^12^. Scheme 4 treated as many individual cranial bones as feasible as separate modules. Scheme 5 was based on Goswami^58^ and Goswami and Polly^17^. Two additional schemes (“Clustering1”, and “Clustering2”) were developed by searching for clusters in the Euclidean distance matrix of the covariance matrix of the Procrustes shape coordinates in the current dataset^21^(e.g., ref. ^58^). 3D covariance matrices were generated in two different ways: (1) using unscaled congruence coefficients of the Procrustes coordinates as applied in the *paleomorph* package version 0.1.4^20^; and (2) using phylogenetically independent contrasts of the Procrustes coordinates and a distance matrix method developed by Griffin^21^. *K*-means clustering was then applied to the distance matrix of the covariance matrix to divide the coordinates into 1 to 30 clusters and a goodness of clustering measure—the gap statistic^59^, which compares cluster assignments against a bootstrapped null model—was used to help determine the optimal number of clusters in the data, using the libraries *cluster* and *factoextra*^60,61^.

In order to reduce variation and covariation between right and left sides of the cranium, all modularity schemes and tests used a symmetrical, one-sided coordinates matrix that included all midline coordinates and lateral coordinates from the right side of the cranium only (by deleting landmarks 23–44). The seven modularity schemes were then assessed using two different (and arguably non-comparable) methods: (1) through maximum likelihood comparisons on a 3D vector correlation matrix of the congruence coefficient of the Procrustes shape coordinates using the *EMMLi* v.0.0.3 package^62,63^; and (2) by testing the variation and covariation of hypothesized modules in a phylogenetic context against a null model using the covariance ratio as implemented in the *geomorph* package^64,65^. The most modular of the seven schemes (that with the highest likelihood value and lowest covariance ratio) was then used for pairwise tests for significant differences in the covariance ratio effect sizes between subfamilies and among tribes.

### Reporting summary

Further information on research design is available in the Nature Research Reporting Summary linked to this article.

## Supplementary information

Supplementary Material

Reporting Summary

## Data Availability

The datasets generated and analyzed during this study, including an R script for performing all analyses, are available via an online repository of the Museum für Naturkunde repository at 10.7479/499j-tv94^66^.
