# Supplementary material for: Evolution of the bovid cranium: morphological diversification under allometric constraint
Source: Commun Biol. 2022 Jan 19;5:69. doi: 10.1038/s42003-021-02877-6 (PMC8770694; doi:10.1038/s42003-021-02877-6)
Supplement: Supplementary file 1 — Supplementary Material [file 42003_2021_2877_MOESM1_ESM.pdf]

Supplementary figures to accompany

Evolution of the bovid cranium: Morphological diversification under allometric constraint

Faysal Bibi*[1]

Joshua Tyler[1,2]

1. Museum für Naturkunde, Leibniz Institute for Evolution & Biodiversity Science, Invalidenstr. 43, Berlin, 10115, Germany

2. Current address: Milner Centre for Evolution, Department of Biology and Biochemistry, University of Bath, Bath, BA2 7AY, United Kingdom

*corresponding author

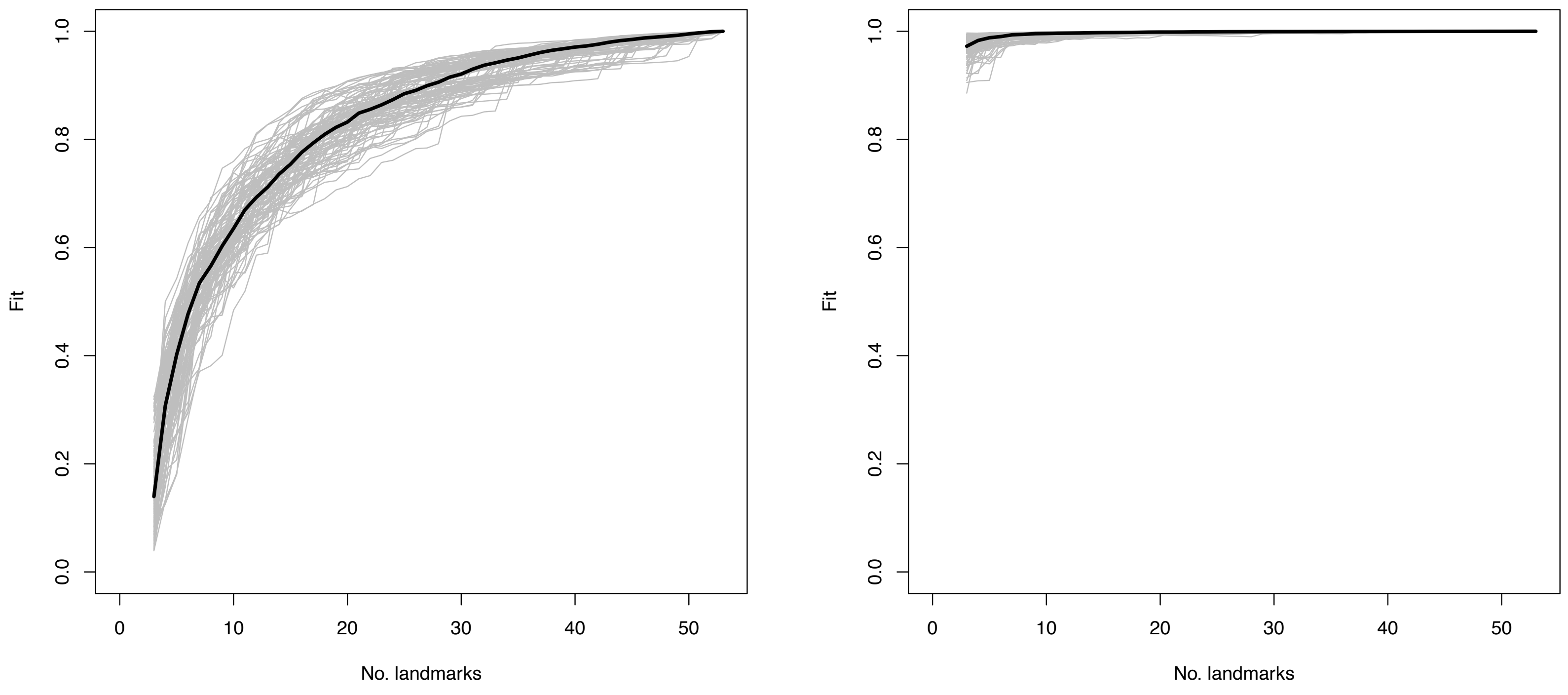

Supplementary Figure 1. Rarefaction curves of shape (left) and size (right) using random subsamples of the total dataset of 53 landmarks (using LaMBDA, Watanabe, 2017). Some 90% of shape variation in our dataset is captured by just 30 landmarks, showing that the number of landmarks used (53) is sufficient.

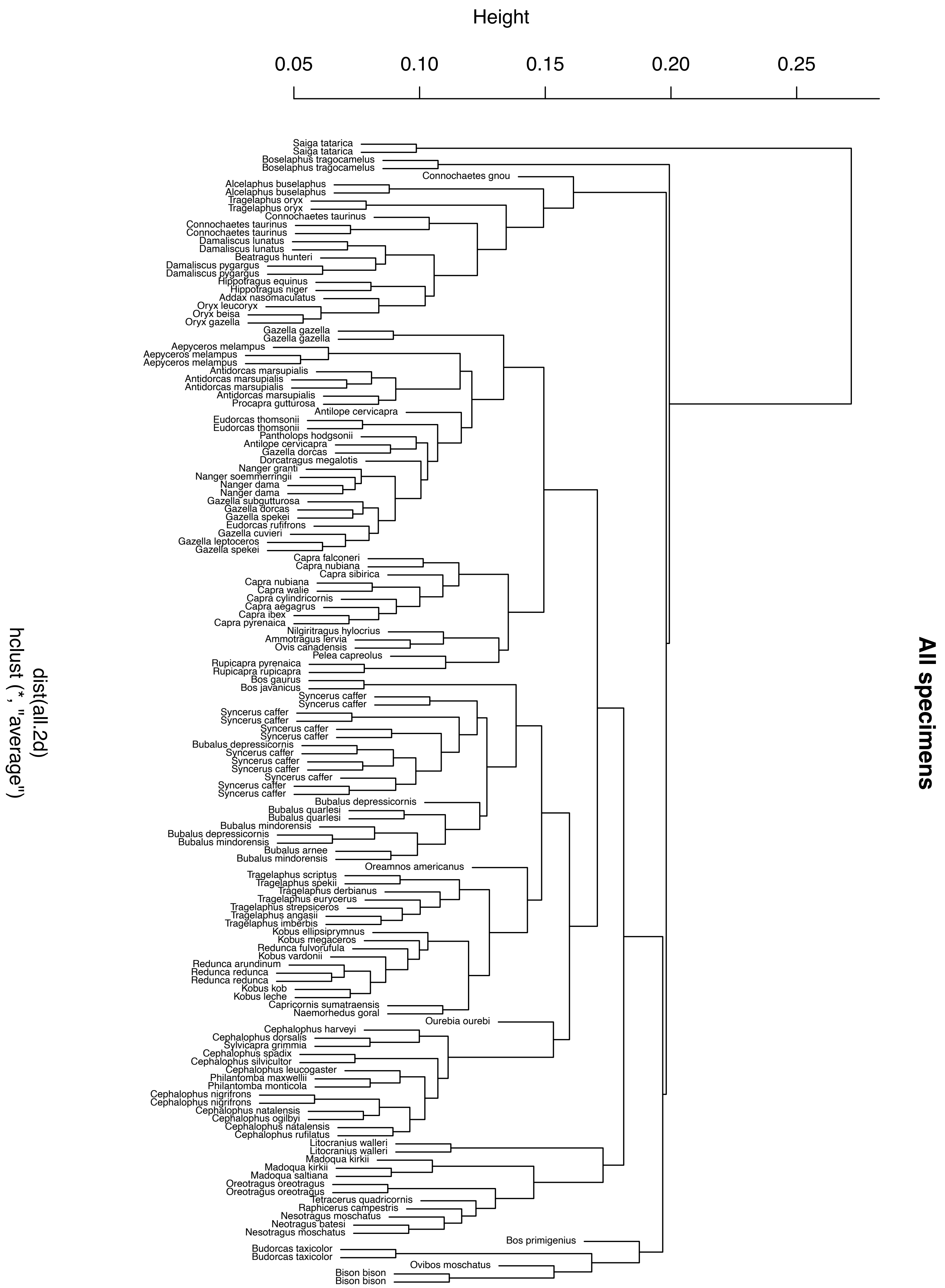

Supplementary Figure 2. UPGMA phenogram of the Procrustes shape coordinates of all specimens. Specimens belonging to the same species mostly cluster together, indicating minor effects of intraspecific variation.

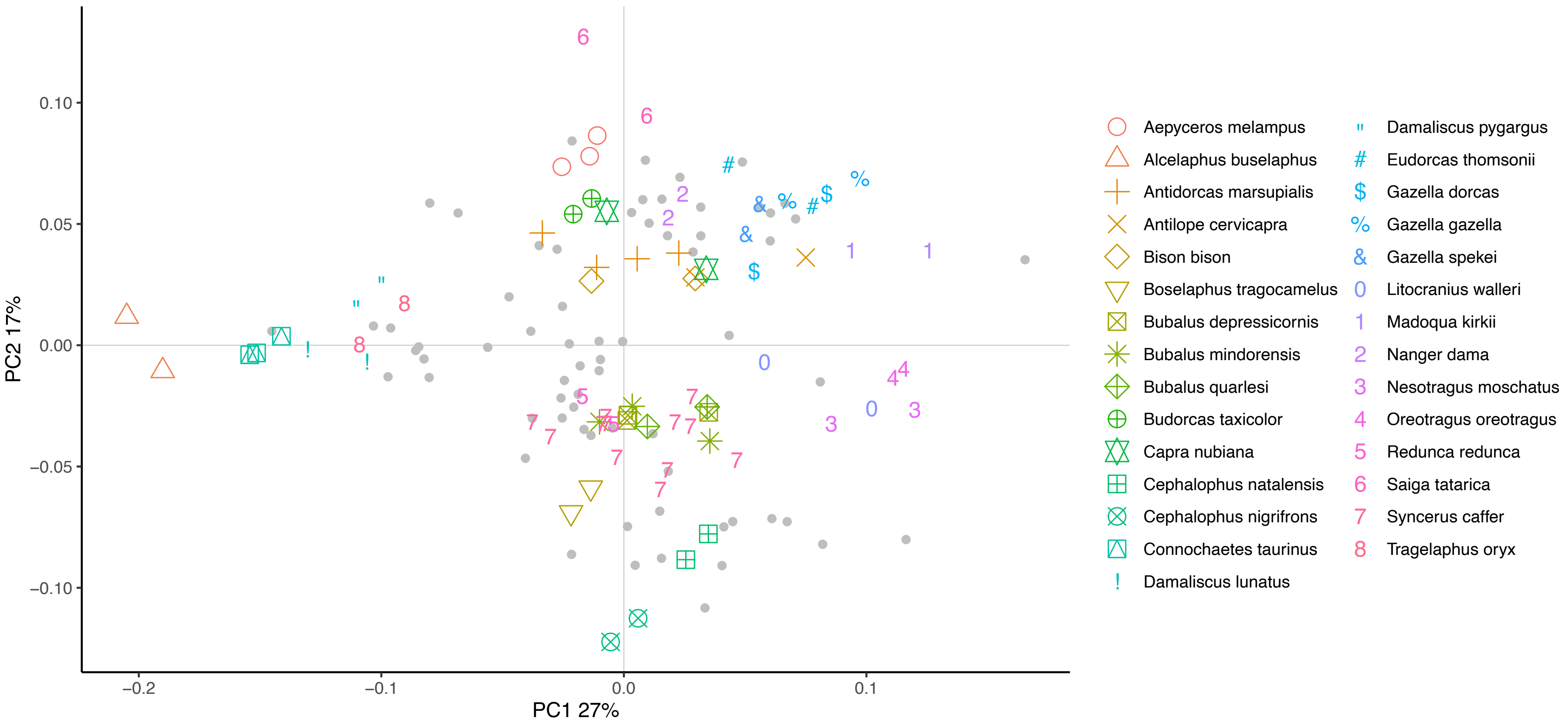

Supplementary Figure 3. The first two principal components of the analysis of all specimens. Crania of the same species are highlighted to show the range of intraspecific variation along these primary components.

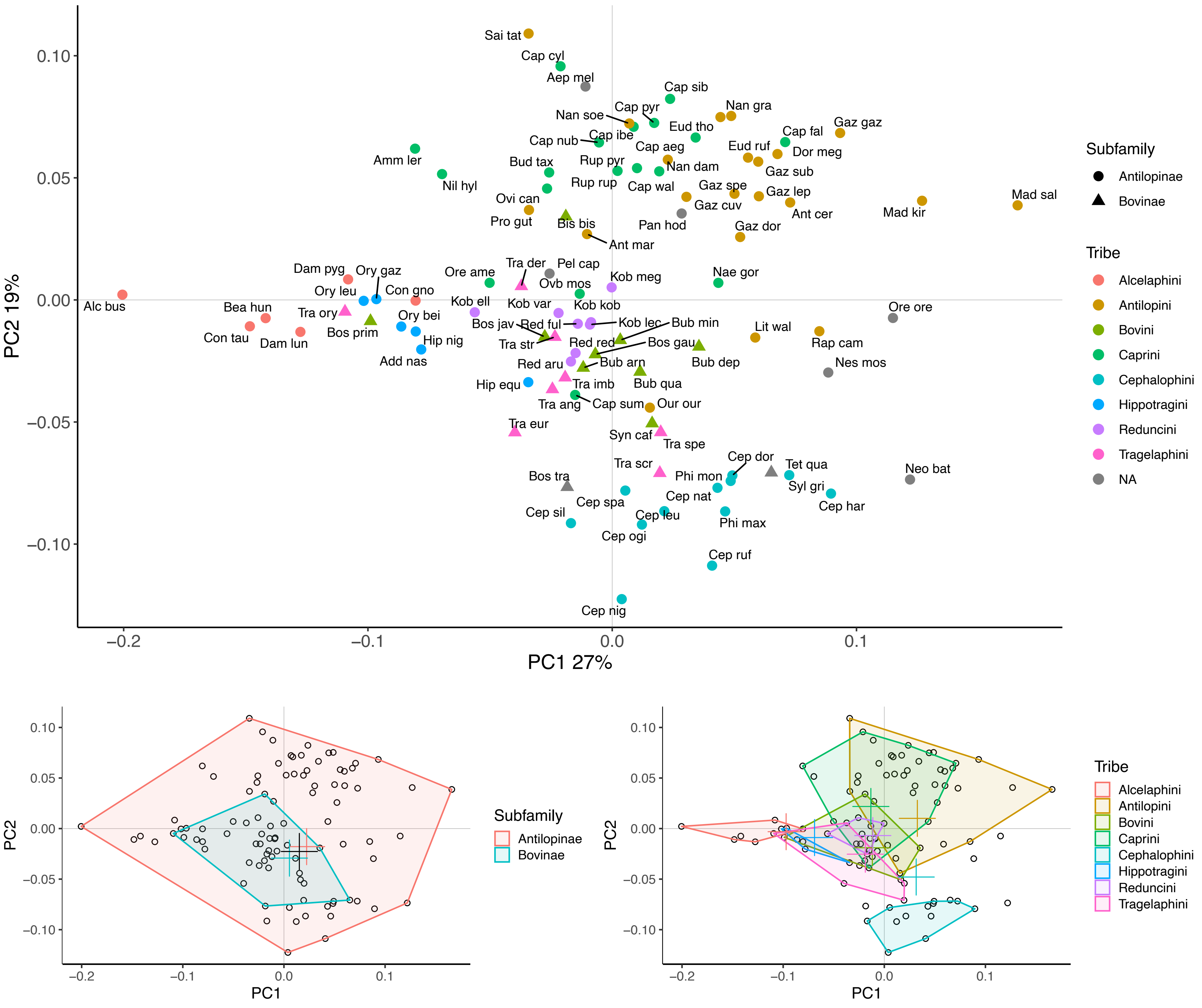

Supplementary Figure 4. PC1 and 2 from the analysis of the phylogenetic subset (one specimen per species). Above, same as Fig. 3 in the main text, but with species labels. See supplementary specimen classifiers spreadsheet for abbreviations. Below, with subfamily and tribe convex hulls. The + symbols mark reconstructed positions of most recent common ancestors of all bovids (black) and subfamilies and tribes.

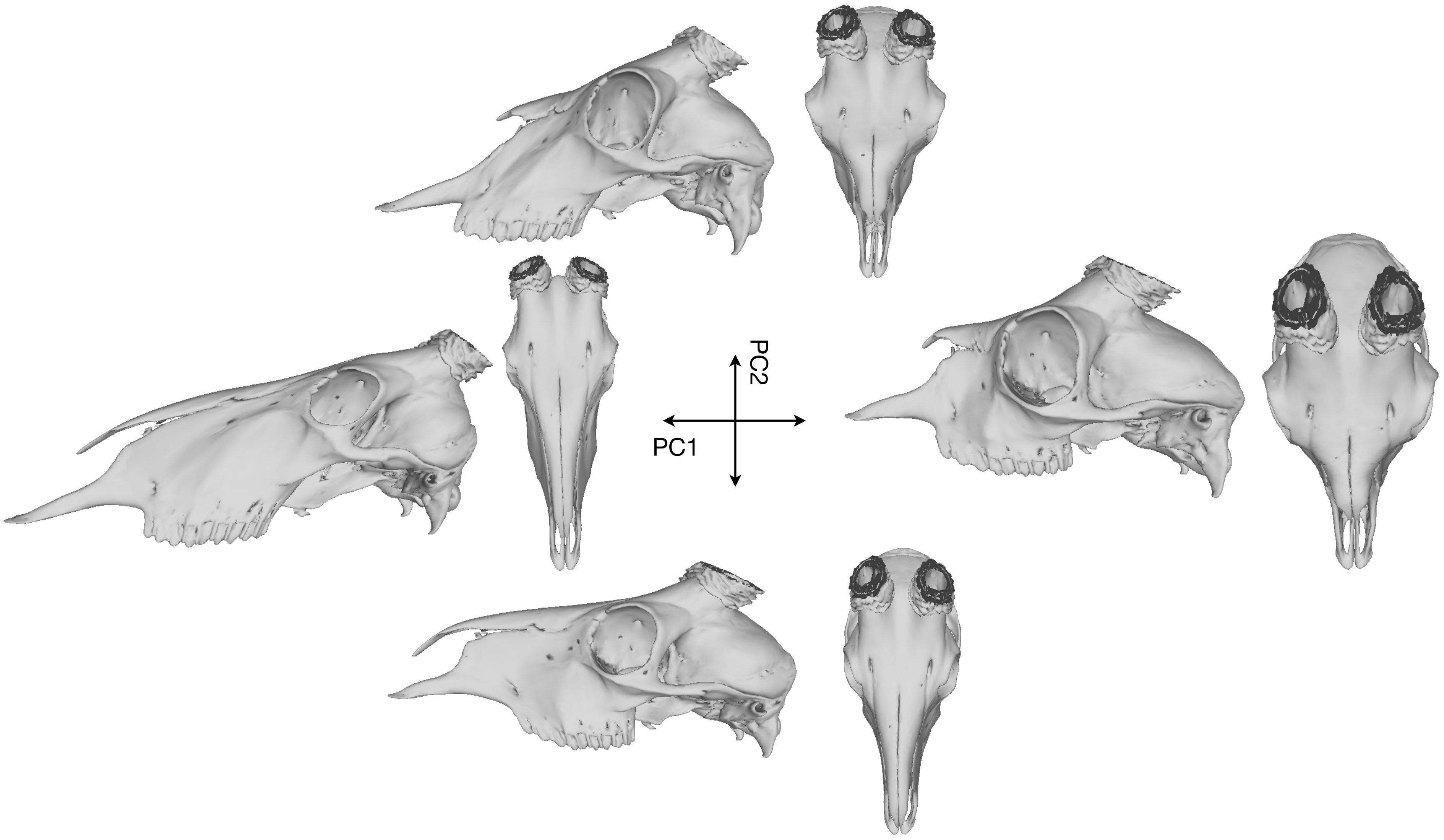

Supplementary Figure 5. Visualization of shape changes along PC1 and 2 in lateral and dorsal views. 3D models made by warping a cranium of *Naemorhedus goral* (AMNH 43033) to the extremes of each PC.

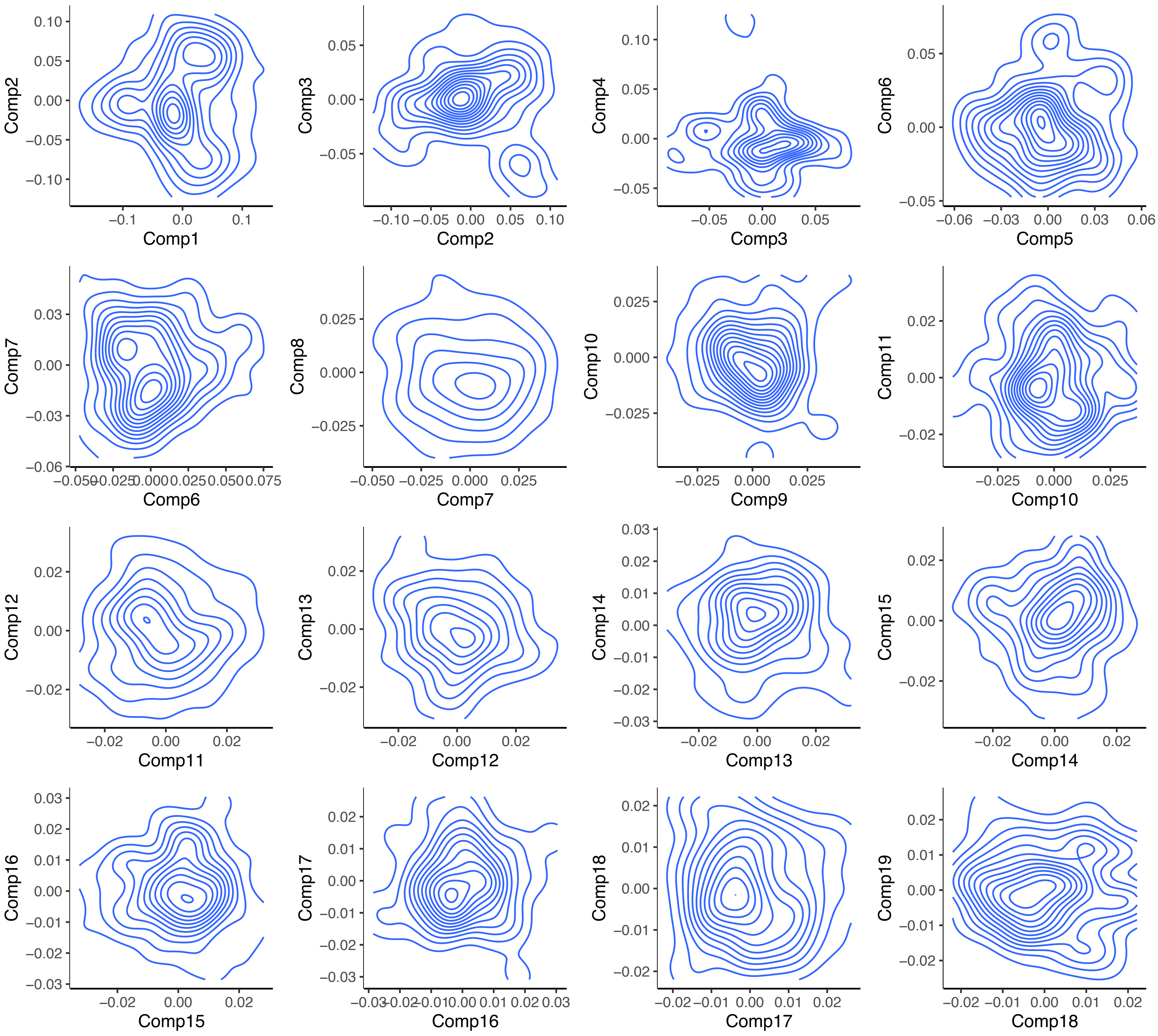

Supplementary Figure 6. Density contours of species occupancy along the first 19 principal components. These show the consistent presence of central clustering (clumping).

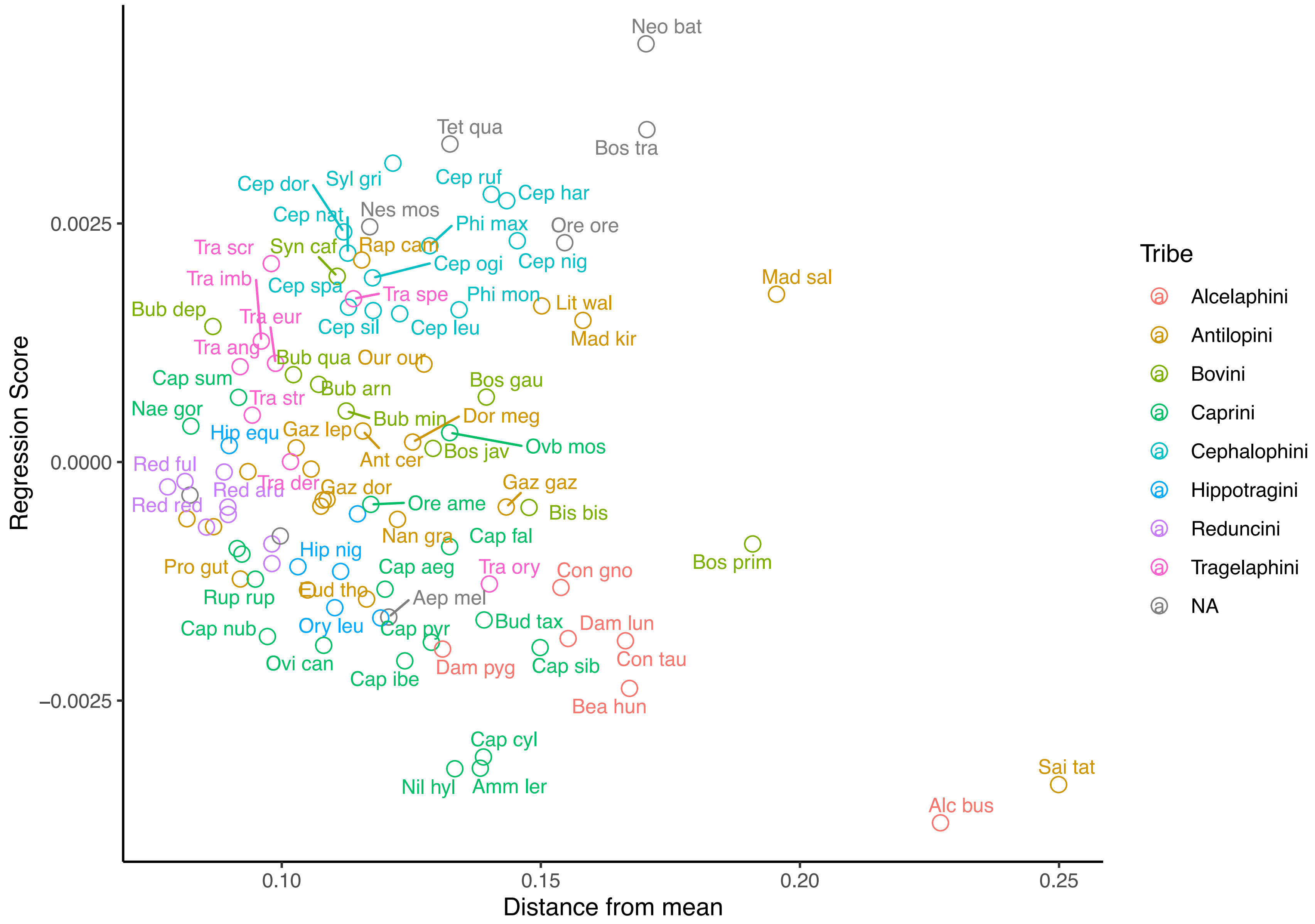

Supplementary Figure 7. Differences in shape in relation to distance from the morphospace mean. Regression score is calculated using the regression of the Procrustes-aligned coordinates on distance from the mean shape. Same as Fig. 5, with species labels.

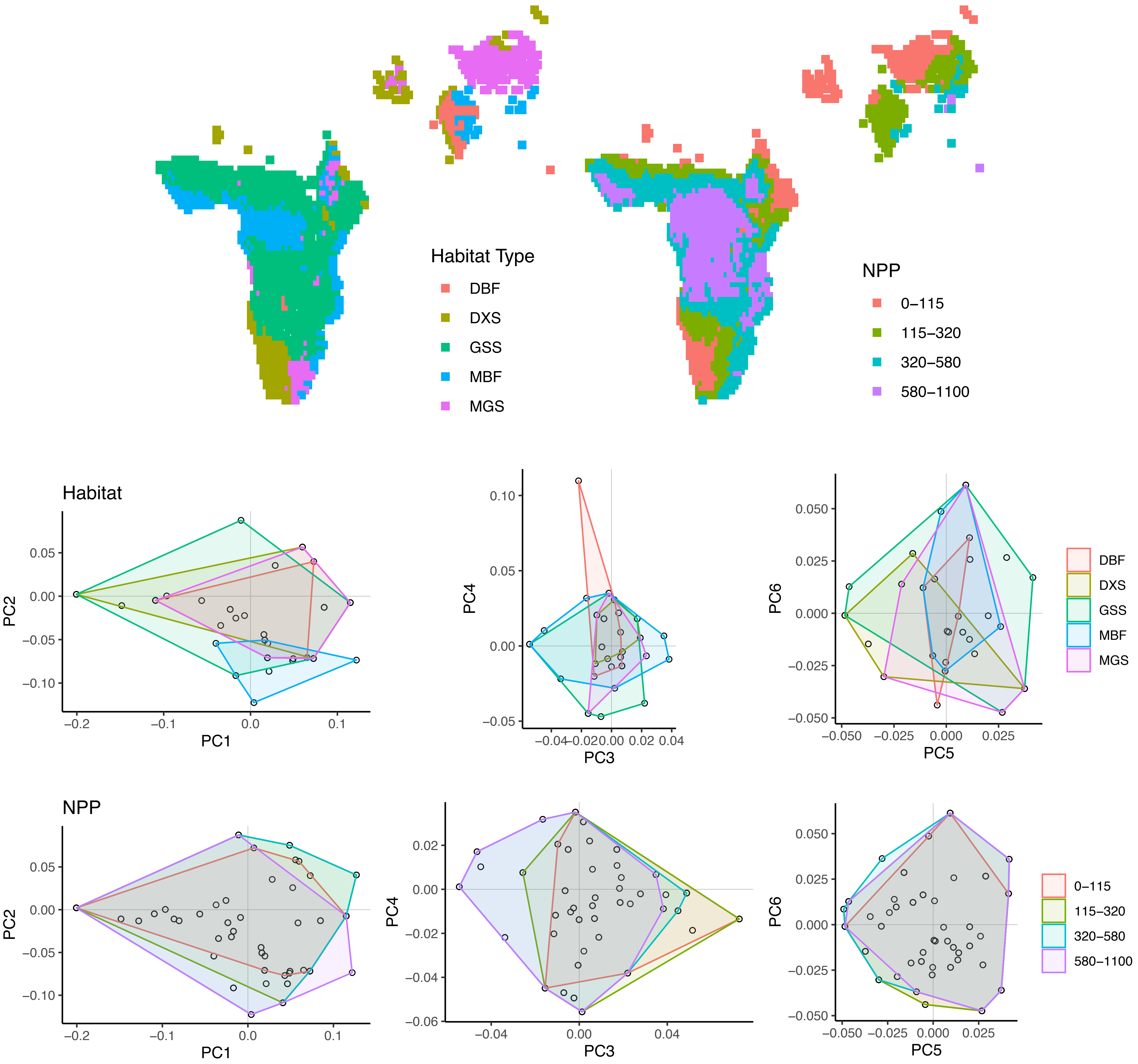

Supplementary Figure 8. Distribution of habitat and NPP categories. Above: geographic distribution of habitats and NPP categories for communities with more than three bovid species. Below: Habitat and NPP occupancy along the first six principal components using the 50% most commonly occurring species. Habitat abbreviations: **DBF**, tropical and subtropical dry broadleaf forests. **DXS**, tropical and subtropical deserts and xeric shrublands. **GSS**, grasslands, savannas, and shrublands. **MBF**, moist broadleaf forests. **MGS**, montane grasslands and shrublands. NPP is in trillions of kgs of carbon per 1 degree square.

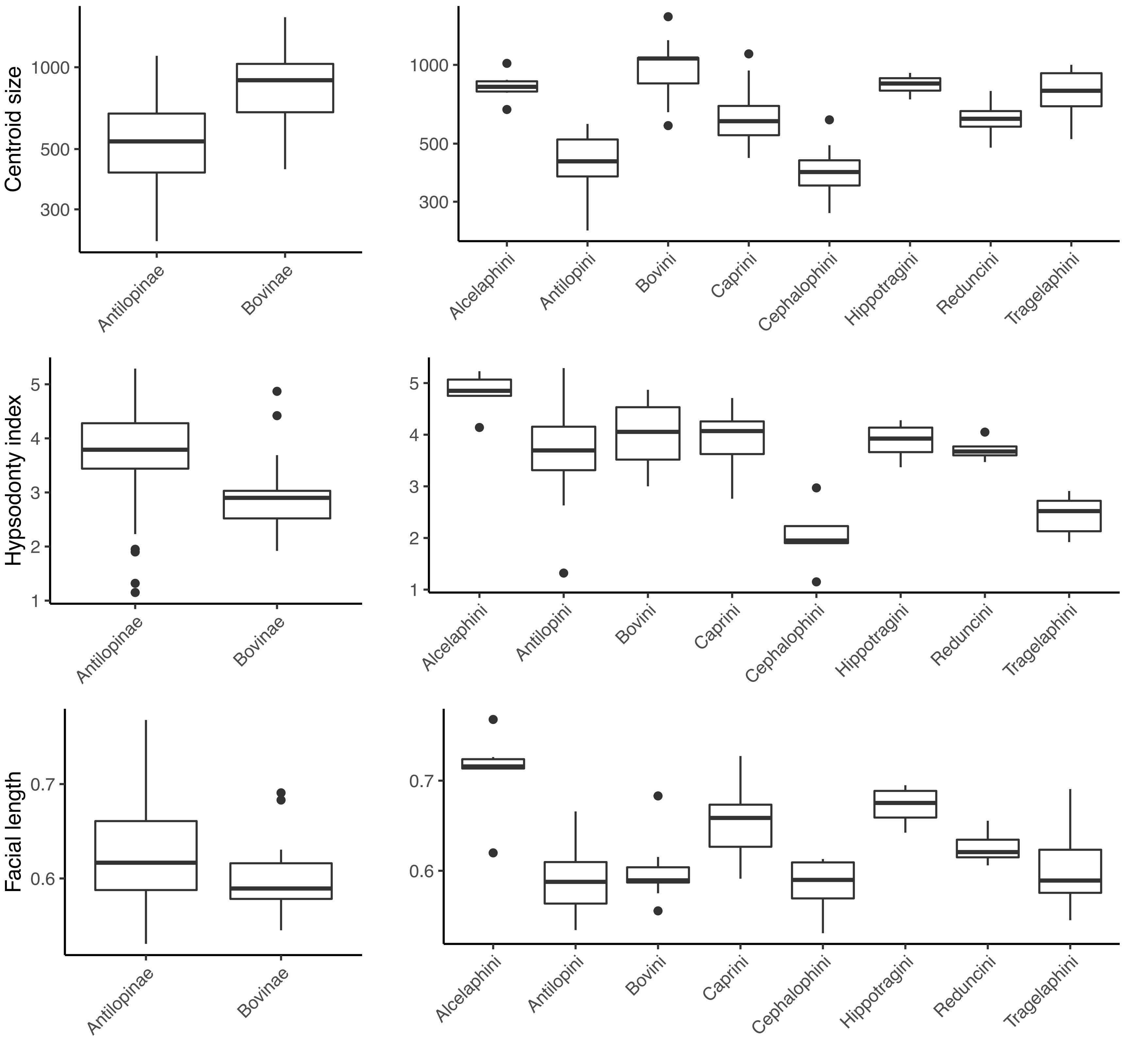

Supplementary Figure 9. Distributions of centroid size, hypsodonty index, and facial length ratio among subfamilies and tribes.

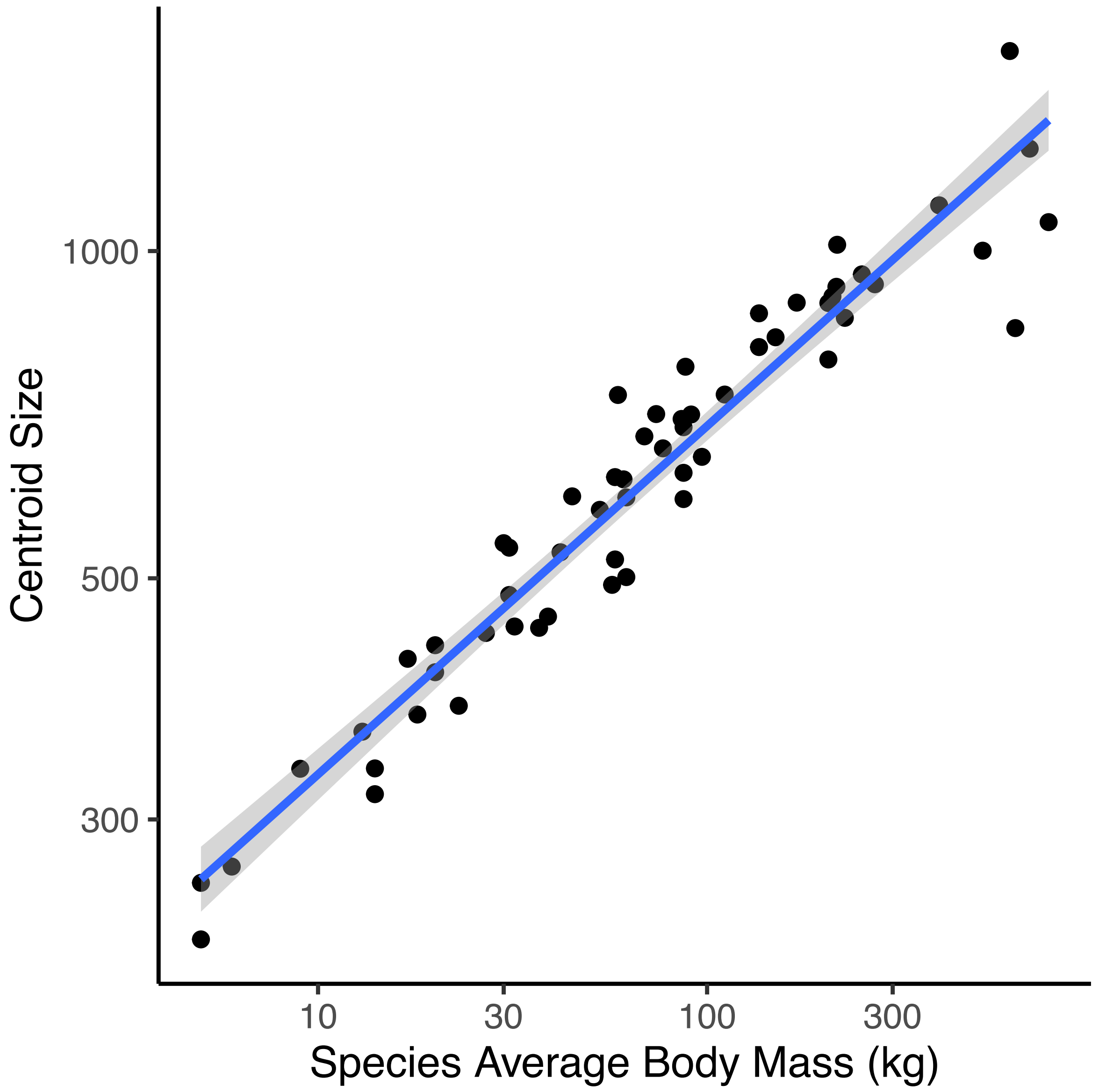

Supplementary Figure 10. Log centroid size shows a strong linear correlation with log species average body mass.

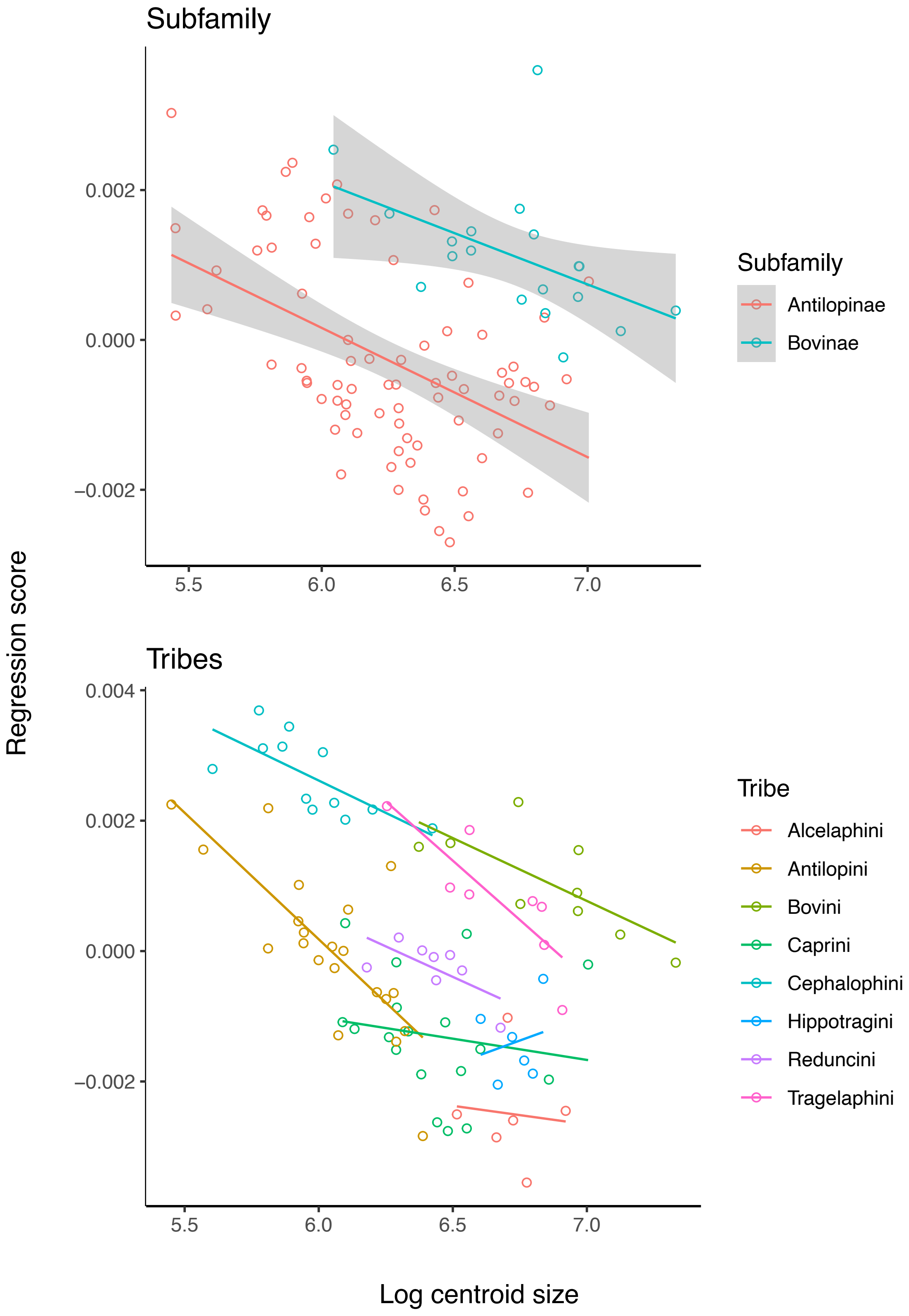

Supplementary Figure 11. The relationship of shape and size by subfamilies and tribes. Shape is represented by the regression of Procrustes-aligned coordinates against log centroid size. The differences in slopes between subfamilies and among tribes are non-significant, indicating similar shape-size allometric trajectories across bovids.

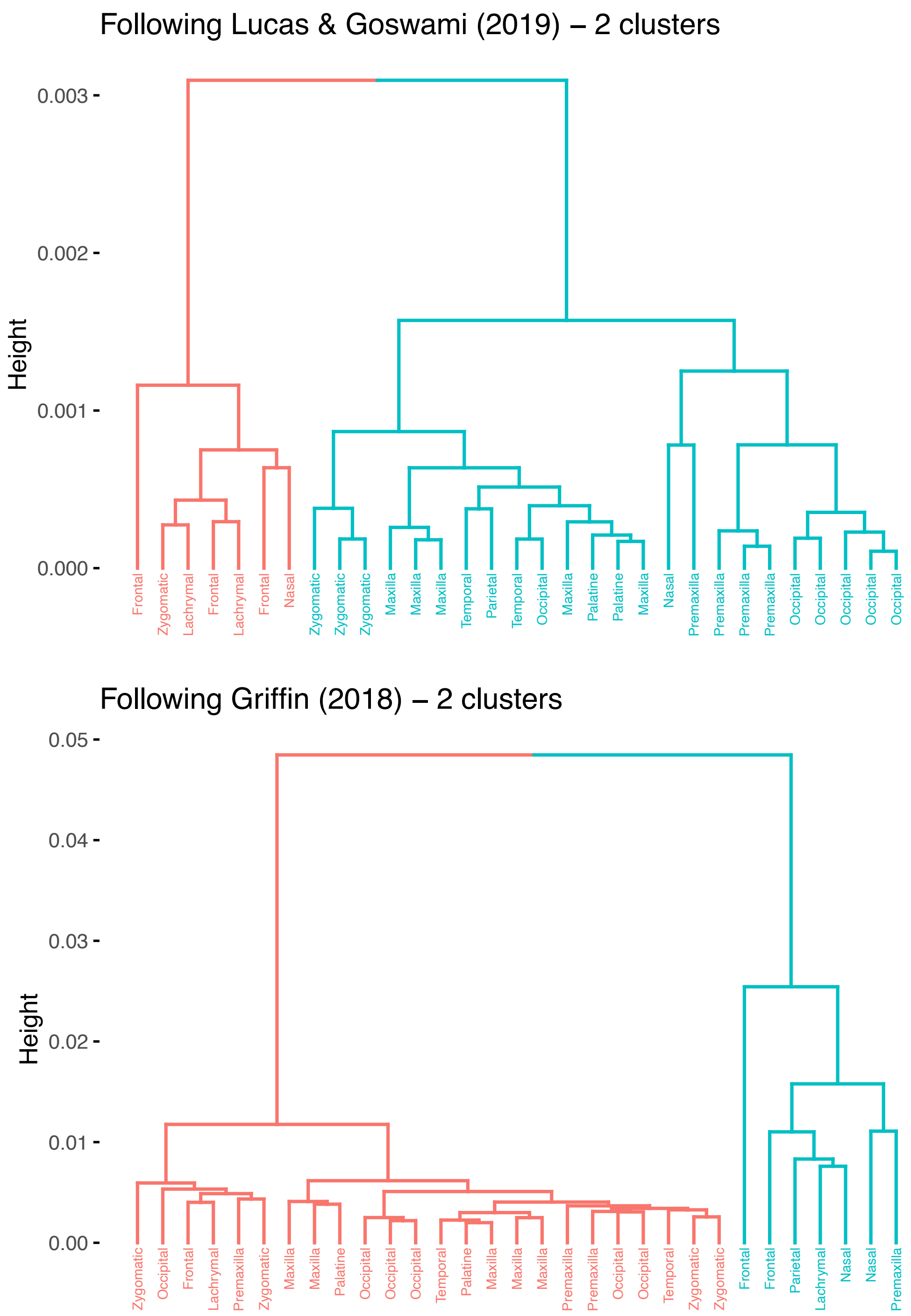

Supplementary Figure 12. Clustering of Procrustes coordinates. Results of dividing landmarks into *k* clusters based on the results of the gap statistic applied to variance-covariance matrices of Procrustes shape coordinates using two different approaches. The Griffin (2018) approach employs phylogenetic contrasts. Both schemes distinguish portions of the orbital or dorsal cranial region from the remainder of the cranium. Coordinates are labelled by the element on which they occur (arbitrarily choosing one element to name sutural coordinates). Note these are a one-sided subset (n=31) of the total (n=53) number of 3D coordinates. The gap statistic favors between 3 to 5 modules for the Griffin (2018) approach, but these would result in one or more modules composed of one or two coordinates only, so instead a more conservative two-module scheme was chosen.

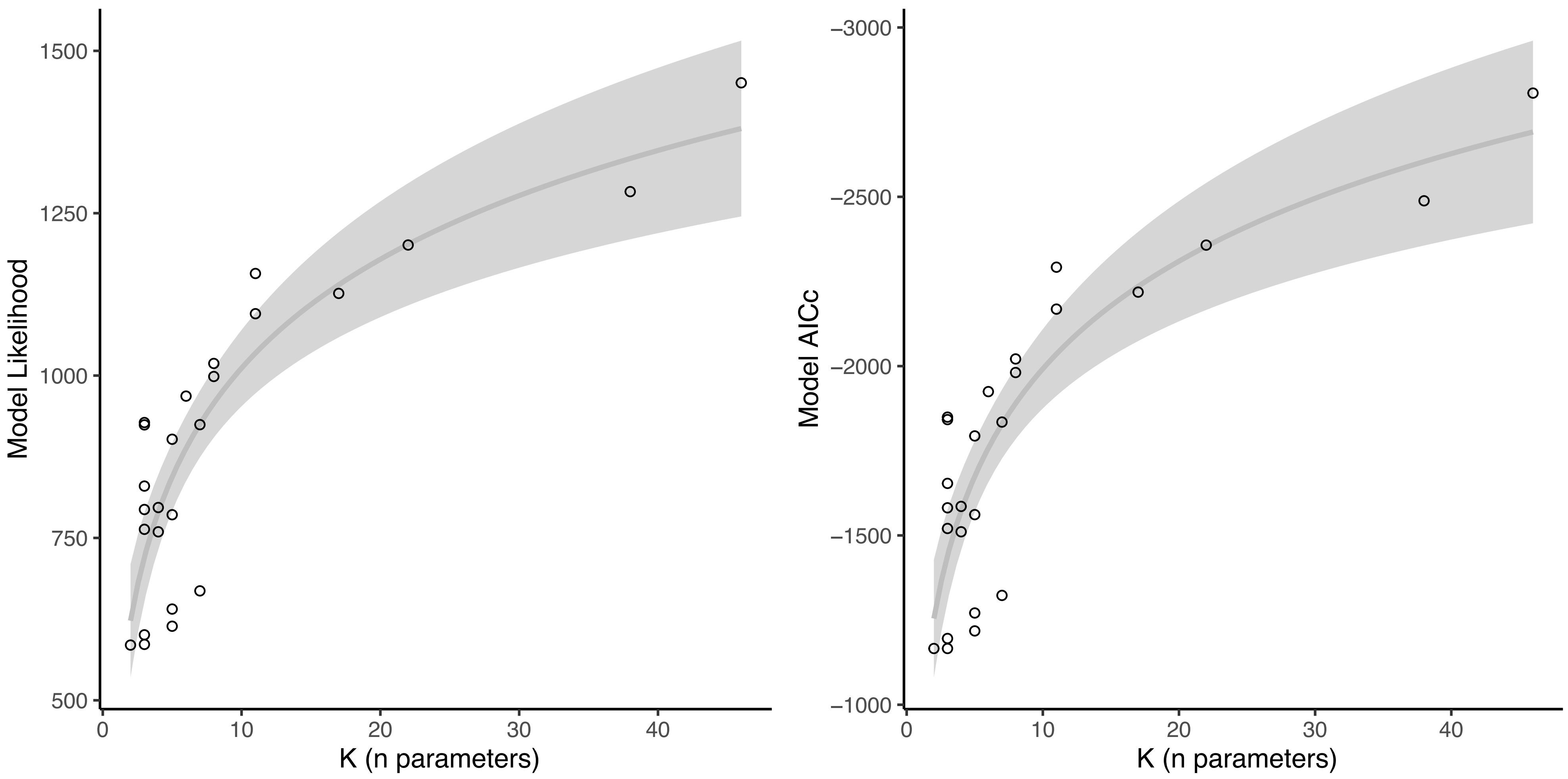

Supplementary Figure 13. Testing modularity schemes using likelihood.
EMMLi (Goswami & Finarelli, 2016) generally favors the most complex modularity scheme, whether assessed using likelihood (left) or AICc (right).
